# A Scoping Review of Stigma Related to Prostate Cancer in Black Men

**DOI:** 10.1007/s40615-024-02070-5

**Published:** 2024-07-09

**Authors:** Olufikayo Bamidele, Sarah Greenley, Blessing Onyinye Ukoha-Kalu, Opeyemi Faith Titus, Veronica Nanton

**Affiliations:** 1 Hull York Medical School, University of Hull, Cottingham Road, Hull, HU6 7RX UK; 2 School of Medicine, University of Nottingham, Nottingham, UK; 3 Nottingham Business School, Nottingham Trent University, Nottingham, UK; 4 Warwick Medical School, University of Warwick, Warwick, UK

**Keywords:** Prostate cancer, Stigma, Black men, Scoping review

## Abstract

**Background:**

Prostate cancer (CaP) disproportionately affects 1-in-4 Black men and is a stigmatised disease within their communities. Yet, Black men are underrepresented in CaP research concerning stigma, which necessitates a scoping review to map available evidence on this topic to inform future research.

**Aims:**

To map published literature on stigma related to CaP in Black men to understand their experiences and/or perceptions and identify directions for future research.

**Methods:**

A scoping review was conducted using the five-step framework by Arksey and O’Malley. Studies published in English addressing stigma related to CaP from the perspectives of Black men and/or their families were included. We searched six databases including Medline, Embase, PsycInfo, CINAHL, Web of Science Core Collection and Google Scholar, from inception to April 2023. Citation searches were also conducted. Two independent reviewers conducted screening and data extraction. Data was synthesised using descriptive content analysis.

**Results:**

Thirty-four eligible studies conducted in the USA, UK, Trinidad and Tobago, South Africa, Cameroon and Canada from 1995 to 2023 were included. A total of 1867 Black men with/without a CaP diagnosis and 145 adult partners were included. Review findings showed a complex intersection of self-stigma, public stigma and structural stigma impacted Black men’s perceptions of their masculinity. While men’s experiences/perceptions of stigma varied depending on their illness status, there were commonalities in their masculinity concerns (underpinned by stigma), which influenced their attitude towards digital rectal examination, post-treatment side effects and social interactions on CaP. These have implications for public health messaging on CaP within Black communities, as well as patient-provider interactions with the men.

**Conclusions:**

This novel review highlights the need to pay attention to how CaP is presented to Black men and their communities using avenues and languages that are culturally acceptable and empower them to negotiate self-stigma, public stigma and structural stigma related to CaP. Directions for further research were also identified.

**Supplementary Information:**

The online version contains supplementary material available at 10.1007/s40615-024-02070-5.

## Background

Prostate cancer (CaP) is the second leading cause of male cancer mortality (after lung cancer) globally, with over 1.4 million cases and 375,000 deaths worldwide in 2020 [1]. In the United Kingdom (UK), CaP is the most common male cancer, affecting over 140 men daily and this incidence is projected to increase by 12% by the year 2035 [2]. While no preventable factor has been associated with CaP, it disproportionately affects 1 in 4 Black men (men of African and/or Caribbean ancestry including Black African, Black Caribbean, Black British and African American) compared with 1 in 8 White and 1 in 13 Asian men [3]. Amidst this disparity, CaP remains a stigmatised disease within Black communities across diverse settings [4–7]. Evidence shows that socio-cultural stigmatisation of CaP among Black communities substantially contributes to delays in help-seeking for early diagnosis. Such delays in help-seeking lead to more advanced stages of CaP at diagnosis [8], poorer survival rates [9], more intensive treatment procedures (leading to complicated and chronic side-effects) [10, 11], and reduced quality of life for men and their partners [12–14].

Little is known regarding what contributes to and constitutes stigma in relation to CaP, particularly among Black men as the majority of available research on stigma and health has predominantly focused on HIV/AIDS [15, 16], obesity [17, 18], leprosy [19] and mental health issues [20]. Findings from these studies show that stigma involves socially constructed negativity towards an individual or a group of people due to their physical, mental or social attributes, including illness conditions. Within the limited literature on CaP in Black men, evidence suggests that stigma may emanate from self, social or interpersonal perceptions of screening procedures (e.g. digital rectal examination) as emasculating [4, 21]; a CaP diagnosis as imminent death [13], post-treatment side-effects (e.g. sexual dysfunction) of CaP as leading to diminished masculinity (e.g. impotence, inability to meet breadwinning obligations) [7] and uptake of psychosocial support as a sign of weakness [22]. However, there remains a very limited understanding regarding the sources of stigma related to CaP and its impact on help-seeking for diagnosis, treatment decision-making, social communications, spousal intimacy, psychosexual support uptake and post-treatment quality of life within the Black cultural context.

A recent review by Larkin et al. [23] sought to evaluate primary stigma domains in relation to patient outcomes and disease management among men of different ethnicities with CaP. However, this was a systematic review of men from different ethnic groups and cited only two studies which reported specific data on Black men. The review reported very limited data which predominantly focused on self-stigma expressed through illness non-disclosure among Black men in the two studies. A comprehensive scoping review that specifically maps wider evidence on stigma within the context of Black men will guide future research in this area. Also noting that stigma is often enacted through social interactions and can vary depending on illness type and socio-cultural context [24], the importance of understanding its impact on the lives of Black men and partners within the CaP context, is sorely needed. Pryor and Reeder [25] developed a conceptual model to enhance understanding of stigma and identified four dimensions: (i) self-stigma, (ii) public stigma, (iii) stigma by association and (iv) structural stigma (Table 1). Evidently, there are complex intersections between an individual’s self-perception and the social identities associated with their circumstances, which may in turn impact their self-esteem, health behaviours and the quality of their relationship with others [26].

**Table 1:** Dimensions of stigma based on Pryor and Reeder’s model [25]

Dimension of stigma	Definition
Public stigma	How people react socially or psychologically to someone they perceive as having a stigmatised condition
Self-stigma	An individual’s acceptance and internalisation of the negative beliefs and social reactions associated with having a perceived stigmatised condition
Stigma by association	People’s reactions to being associated with a person with a stigmatised condition as well as the social and psychological reactions they receive from others because of their associations with a stigmatised person (e.g. spouse, family, friends, marriage)
Structural stigma	The society’s perpetuation of a stigmatised belief, attitude or person through institutional, cultural and systemic ideologies that legitimises perceptions of a stigmatised status

Therefore, this scoping review aimed to (i) map the current state of evidence on stigma related to CaP in Black men and (ii) contextually understand Black men’s experiences and/or perceptions of stigma related to CaP. Noting the dearth of research in this area, this review would also help to identify specific directions for future research. Such future research would be essential to inform the development of evidence-based, tailored and strategic educational interventions which would be personally, socially and culturally acceptable to tackle stigma and improve the knowledge, attitude and practices towards CaP among Black men and their communities.

## Methods

### Study Design

The review was conducted using the five-step framework recommended by Arksey and O’Malley [27]: (1) identifying the research question; (2) identifying the relevant studies (defining the inclusion and exclusion criteria); (3) searching and selecting the evidence; (4) charting the evidence and (5) collating, summarising and reporting the evidence. The review protocol was published on the Open Science Framework (OSF) website (https://osf.io/k5ptc.). The review is reported in line with the Preferred Reporting Items for Systematic Reviews and Meta-Analyses extension for Scoping Reviews (PRISMA-ScR) Checklist [28].

#### Identifying the Research Question

The review addressed two research questions (i) what is the current state of evidence on stigma related to CaP in Black men? and (ii) what are the experiences and/or perceptions of stigma related to CaP among Black men?

#### Identifying Relevant Studies (Defining the Inclusion and Exclusion Criteria)

Relevant studies were identified using defined inclusion and exclusion criteria as guided by the PCC (Population, Concept and Context) framework for non-intervention studies [29] (Table 2). Regardless of their methodological designs, studies were included if they were published in English language and (a) reported on perceptions, beliefs or experiences of stigma among adult Black men, their partners or family members in relation to CaP and (b) contextualised stigma related to CaP (e.g. around the early presentation of symptoms, help-seeking for early diagnosis, screening uptake, treatment decision-making, spousal communication, social interactions, uptake of post-treatment support and coping and social support). Studies which lacked methodological detail or empirical data (such as commentaries and editorials) and non-English studies were excluded (due to non-availability of resources for transcribing).

**Table 2:** PCC framework for selection of studies

	Inclusion criteria	Exclusion criteria
Population	(i) Adult Black men who are either healthy or diagnosed with CaP (ii) Adult partners or family members of Black men Note: Black men refers to those of African ancestry including Black African, Black Caribbean, Black British, African American, Black Mixed	Adult men of any other ethnicity apart from Black
Concept	Perceptions, beliefs or experiences of stigma (expressed by study participants in different ways either self-stigma, public stigma, stigma by association or structural stigma) in relation to CaP	Perceptions, beliefs or experiences of stigma in relation to palliative or end of life care
Context	Studies published in English Language and of any publication date which contextualise stigma associated with CaP along the entire illness spectrum (pre-diagnosis, diagnosis, treatment and post-treatment), as follows: (i) Early presentation of symptoms (ii) Help-seeking for early diagnosis/screening procedures (iii) Treatment decision-making (iv) Spousal communication (v) Social interactions on the CaP topic (vi) Illness disclosure (v) Access and utilisation of post-treatment support and (vi) Coping and social support	Non-English studies

#### Searching and Selecting the Evidence

A comprehensive search was conducted on a range of sources likely to retrieve relevant material for our research questions based on early scoping. These include the large biomedical databases Medline All (from 1946) and Embase (from 1974) both via OVID, more specialist databases covering behavioural and social sciences (PsycINFO from 1967) via OVID and nursing and allied health (CINAHL Complete via EBSCOhost, multidisciplinary indexes [Web of Science Core Collection, including SCI-Expanded, SSCI, Arts and Humanities Citation Index, Conference Proceedings Citation Index, Emerging sources via Web of Science) and Google Scholar. Grey literature (reports and unpublished studies) was identified by conducting additional searches on Prostate Cancer UK and Movember websites and consultation with professional colleagues. The database search was developed initially in OVID Medline by an experienced information specialist (SG) by examining relevant items potentially meeting the review’s criteria retrieved during a focused search and a previous review [22]. Forward and backward citation searching was performed: the reference list of included studies and related reviews was hand-searched and forward citations of included studies were identified using Citation Chaser software [30]. Initial searches were completed on 05–07 July, 2022. Databases were searched from their inception dates and no language or date limits were applied. An update search for all databases was conducted on 03 April 2023 with results limited to items added to each since the initial searches were conducted.

The search development process tested the combination of four main search concepts (Black men, prostate cancer, stigma, sexual dysfunction) and their indexed and free-text synonyms to retrieve relevant studies. As early testing revealed the possibility of missing studies relevant to our population (e.g. where ethnicity was not explicitly stated in the abstract or indexed terms but a sub-group analysis was provided in the full paper), we used a multi-component search. As early scoping searches indicated that there was very little research published before 1990 on the concepts of prostate cancer AND stigma, our search did not explicitly include outdated terms for Black people. After testing and refinement of the Medline search strategy and peer review by another team member (OB), this was translated for use in other databases. Details of the database and supplementary searches are provided in Appendix 1. Search results were downloaded into Endnote bibliographic software and deduplication was undertaken using a systematic method [31]. The search is reported according to PRISMA-S extension [32].

**Fig. 1:**
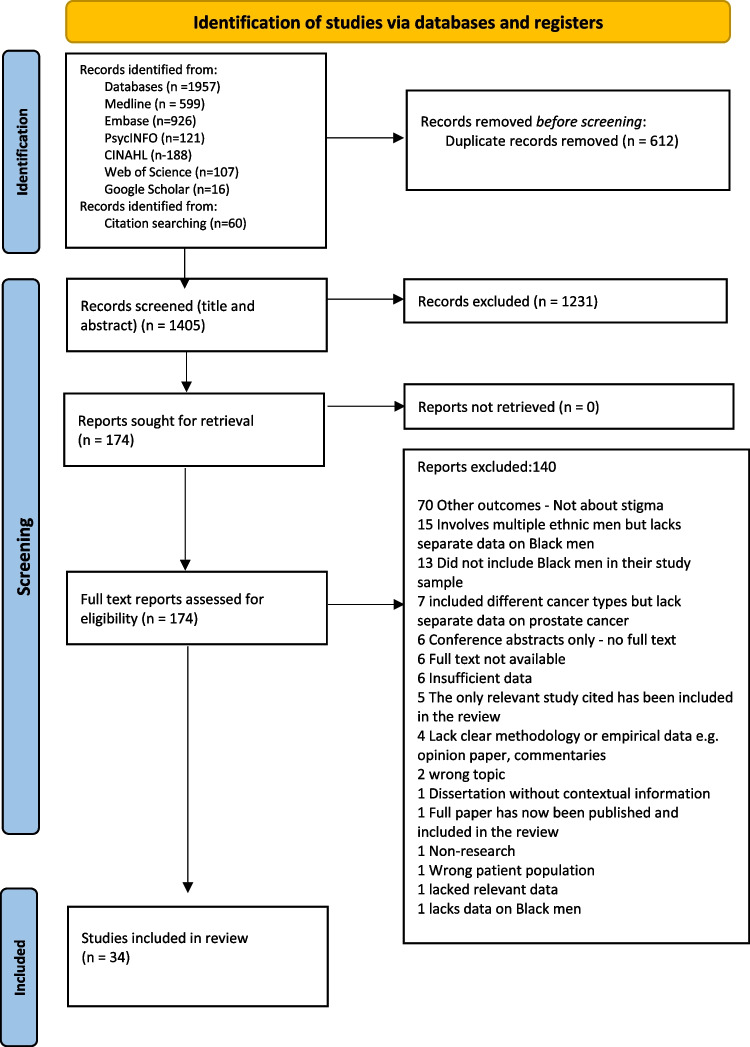
PRISMA flow of study selection

Following the removal of duplicates, the remaining records were uploaded into Covidence software [33] for screening to enhance transparency. The titles and abstracts of retrieved studies were independently screened by at least two reviewers (OB with SG, BUK or VN) to remove irrelevant articles. Full text of potentially relevant articles was then screened against the review’s inclusion and exclusion criteria. Differences in opinion were resolved through discussion to reach a mutual agreement. The study screening and selection process is reported according to the PRISMA-ScR guideline [28] (Fig. 1).

#### Charting the Evidence

Data from the included studies were extracted as relevant to the review aims and presented in Table 3.

**Table 3:** Characteristics of included studies (*n* = 34)

Authors/year of publication	Country	Study aim	Study design	Sample	Perceptions/experiences of stigma in relation to prostate cancer	Context of stigma related to prostate cancer	Dimension of stigma (as mapped to the Pryor and Reeder (2011) conceptual model)	Study limitations	Directions for future research
Ahiagba et al. 2017 [58]	UK	To identify and explore factors that may influence black men and their significant others’ knowledge and awareness of prostate cancer screening	Literature review (7 studies included), thematic synthesis	302 Black men (with and without a prostate cancer diagnosis) and partners; aged 18 years and above	Perception of stigma in relation to undergoing digital rectal examination; fear of being diagnosed with prostate cancer due to cultural taboos around the subject	Social interactions; screening procedure;	Self; structural	Inconsistency in data extraction from the included studies especially regarding the demographics of the participants. All the studies were conducted in the US except one conducted in Barbados	Need for more UK-based research on the perceptions and knowledge of prostate cancer screening among Black men and their significant others
Allen et al. 2007 [39]	USA	To explore African-American (AA) men’s perceptions about prostate cancer screening and assesses the acceptability of various strategies and settings for interventions to promote informed decision-making	Qualitative research using focus groups and in-depth interviews	51 AA men aged 35 –70 years—[healthy men (*n* = 37) and prostate cancer survivors (*n* = 14); Most had completed high school (81%) and had income of $45 K or less	Perception of cancer as a taboo subject to be avoided in public discussions; perception of cancer diagnosis as a death sentence and hospitals as “places where you go to die”	Social interactions; decision-making for diagnosis	Structural; public	Study conducted in one US state; potentially biased sample of men who may already be inclined to positive healthcare behaviour	Need for further studies to develop and evaluate culturally competent interventions to educate and guide informed decision-making for prostate cancer service among Black men
Bamidele et al. 2019a [59]	UK	To explore the psychosocial experiences of Black African and Caribbean men with prostate cancer and their partners in the UK as they lived through the side effects of treatment within their socio-cultural and marital contexts	Grounded theory using interviews and focus groups (HCPs only)	25 Black men (African/Caribbean) aged 75–84 years treated for prostate cancer, 11 partners aged 38–74 years and 11 HCPs within the urooncology team	Perceived cultural stigmatisation of prostate cancer as linked to men’s sexual lives which is a private aspect not to be discussed publicly; cultural expectation for men to be stoic and avoid discussions on their sexual inadequacies	Social interactions; masculinity concerns; socio-cultural masculine expectations	Self; structural; public	The influence of acculturation to a culturally different UK society on men’s experiences is unclear in the study	Need for larger studies to explicate heterogeneity within the Black African and Black Caribbean ethnic groups and identify how much of their prostate cancer experiences can be attributed to age, marital status, ethnicity and acculturation
Bamidele et al. 2019b [60]	UK	To: (a) report the strategies used to recruit Black African and Black Caribbean men with prostate cancer and their partners into a grounded theory study; (b) discuss the barriers and facilitators to recruitment and (c) provide useful suggestions for other researchers seeking to engage similar groups and other “hard to reach” populations in their studies	Case study of recruitment strategies used in a qualitative study of BA and BC men with prostate cancer and their partners	25 Black men (African/Caribbean) aged 75–84 years treated for prostate cancer, and 11 partners aged 38–74 years	Perceptions of stigma associated with prostate cancer within the BA and BC communities influenced illness non-disclosure and limited recruitment into the research	Social interactions; research participation	Self	Actual response rate for the study could not be assessed because the overall number of participants who were approached were not recorded by the HCPs	Need for future studies to explore from gatekeepers’ perspectives, their experiences with researchers and the challenges of promoting research participation among their Black African and Black Caribbean groups with cancer
Bamidele et al. 2022 [62]	UK	To synthesise findings from published studies on the barriers and facilitators to accessing and utilising post-treatment psychosocial support by Black men after prostate cancer treatment	Systematic review and qualitative synthesis	139 Black men (60 AA, 60 BC, 18 BA and 1 unspecified) aged between 49 and 85 years were included	Cultural stigmatisation of masculine sexual dysfunction after prostate cancer treatment was a barrier to illness disclosure and accessing post-treatment support	Masculinity concerns; decision-making for post-treatment support; social interactions	Self; public	Included studies were conducted in the UK (5), USA (4) and Canada (1) with different healthcare structures and contexts. It is unclear how men’s support needs evolved through the post-treatment phase as included studies did not detail length of time since treatment	Need for psychosocial intervention studies focused on behavioural issues among Black men with prostate cancer
Blocker et al. 2006 [38]	USA	To explore the knowledge and beliefs of African-American (AA) men and their spouses about prostate cancer, behaviour change to reduce prostate cancer risk and prostate cancer screening as well as barriers to making health promoting lifestyle changes	Qualitative study (focus groups)	14 AA men and 15 AA women aged 34–68 years old mostly recruited from the church (91%) and mostly married (95.5%)	Embarrassment/shame and fear of “assault on manhood” by having a digital rectal examination; fear of impotence and the associated socio-cultural stigma if diagnosed with prostate cancer	Masculinity concerns; treatment side-effects	Self; Structural; Public	Findings may not be generalisable to other AA and non-AA populations, other geographic areas and to people not attending churches. Men may have been inhibited in sessions moderated by women	Need for tailored prostate cancer interventions, which are culturally relevant to AA churchgoers
Demark-Wahnefried et al. 1995 [37]	USA	To document the characteristics of 1504 men who reported to Prostate Cancer Awareness screening events at nine major sites in 1992 and to report differences in prostate cancer-related knowledge, beliefs and screening behaviour between Blacks and Whites	Self-administered Survey	1504 White and Black men (20% were Black, *n* = 300—78% of the Black were married	Embarrassment associated with digital rectal examination; Taboo around disclosure as significantly fewer Black men (38% vs 51% in white men) report that they have ever known someone with prostate cancer	Social interactions; screening avoidance	Self; structural	Self-selected population of men choosing to attend screening: may not be generalisable	Need for bigger studies to determine the efficacy of prostate cancer screening
Evans et al. 2005 [69]	Canada	To explore the cultured and gendered dimensions of African Nova Scotian men and women’s experiences of breast and prostate cancer	Qualitative study (focus groups using participatory action research)	57 African Nova Scotian and 4 White participants; first phase focus groups included 51 (16 men and 35 women), second phase 21 people (8 men and 13 women) with 6 new to the study	Sense of shame or stigma associated with illness that "pertains to parts of the body “you don’t really want to reveal to people” such as the breast and prostate; perception of diminished masculinity from post-treatment sexual dysfunction prostate cancer leading to shame and embarrassment; Avoiding digital rectal examination due to masculinity concerns	Masculinity concerns; decision-making for diagnosis; screening avoidance	Self	Findings on a small group of men and women from the African Nova Scotian community—not generalizable	Additional research is needed to explore the relationship between gender, cancer and health seeking behaviours, the social construction of masculinity/femininity in African Nova Scotian communities and health inequality and intersection of gender, race and class
Friedman et al. 2009 [43]	USA	to explore the implications of applying a multidimensional health literacy framework to AA men’s understanding and knowledge about prostate cancer	Two-phased study involving survey (phase one) and interviews and focus groups (phase two)	AA men aged 47–64 years with no history of prostate cancer, mostly unmarried (52%)	Perception of prostate as a generational taboo which is not publicly talked about for “fear of being perceived as weak”; cultural expectation for men to be stoic and avoid discussing their health concerns	Masculinity concerns; social interactions; socio-cultural masculine expectations	Structural	A purposively selected sample from a local service agency which may not accurately represent the perceptions and opinions of AA men who are not clients of the agency or who reside elsewhere	There is need for intervention studies to develop and test culturally relevant messages for AA men
Friedman et al. 2012 [47]	USA	To explore AA men’s and women’s current practices, barriers and recommended strategies for prostate cancer communication	Focus group with demographic questionnaire	43 AA men and 38 AA women) aged 21–77 years (mean age men: 52 years; mean age women: 50 years)	Perception of shame and embarrassment associated with digital rectal examination and post-treatment impotence leading to avoidance of public discussion of prostate cancer and feelings of diminished sexuality/masculinity	Treatment side effects; screening avoidance; masculinity concerns; social interactions	Self	A self-selected convenience sample from one southern U.S. state are not representative of entire AA populations	Need for feasibility studies to (i) train both AA women and clergy to educate AA men about prostate cancer and (ii) develop interventions which consider a team approach to decision-making
Fyffe et al. 2008 [42]	USA	To examine underserved black males’ perspectives about prostate and colorectal cancer screening	Qualitative study using the focus group	24 adult Black men with mean age of 53.2 years old, mostly unmarried (83.3%) without a cancer diagnosis	Sensitivity to rectal examinations because of link to men’s perceptions of their sexuality and manhood. Avoidance of prostate and colorectal cancer screening due to stigma and embarrassment associated with DRE. Reluctance towards social interactions on prostate screening for fear of being perceived negatively by their peers	Masculinity concerns; social interactions; screening avoidance	Self; structural; public	Lack of clear additional information that distinguished between prostate and colorectal cancer screening	Need for larger studies to examine the relationship between the demographic characteristics of participants in relation to their positive and negative responses
Griffith et al. 2007 [40]	USA	To understand how the structural environment, affect rural, southern African American (AA) men’s decision-making regarding prostate cancer screening and treatment	Qualitative study using focus group	66 AA men aged between 35 and 83 years	Shame associated with feelings of diminished masculinity due to loss of sexual function post-treatment	Masculinity concerns; treatment side effects	Self	Findings are unique to the rural, southern AA men sample and are not necessarily generalizable to other AA men	Exploration of the influence of the structural environment on informed decision-making regarding prostate cancer screening and treatment. Examination of the unique and additive effects of race, socioeconomic position, region and urban city, and the health protective and health risk norms within AA communities that can be effectively altered to produce desired outcomes
Guan 2023 [55]	USA	To investigate treatment decision-making in a diverse population of patients diagnosed with low- and very-low risk prostate cancer, with a special focus on reporting differences and similarities in sociocultural factors across racial and ethnic groups	Qualitative study involving semi-structured interviews	43 prostate cancer patients with mean age of 61.2 years and of different ethnicities—Asian American (*n* = 13); Black (*n* = 10), Hispanic/Latino (*n* = 10); White (*n* = 10), mostly married or living with partner (79.1%)	Socialised expectations of masculinity led to feelings of humiliation from treatment side-effects such as incontinence and sexual dysfunction. Fear of losing idealistic masculine expectations influenced choice and decision-making for treatment	Masculinity concerns; socio-cultural masculine expectation; decision-making for treatment; treatment side effects	Self	Patients had already completed the decision-making process prior to participation in the study, hence findings may not capture factors immediate arising during the process of making treatment decisions	Future studies should investigate disparities in the masculinity norms based on sexual orientation, gender identity, relationship preferences and how these intersect with managing a prostate cancer illness
Harvey et al. 2011 [46]	USA	To explore and understand the contextual factors in the attitudes and beliefs of African-American men’s view of health in general, and as related to prostate cancer in particular	Qualitative study using focus group	15 African-American men with an average age of 56.9 years mostly married (87%) and without a prior diagnosis of prostate cancer	Perceived DRE as a violation of manhood and indicative of homosexuality which men were uncomfortable with and regarded as culturally unacceptable	Screening avoidance; masculinity concerns; sexuality concerns	Self; structural	A relatively small sample (*n* = 15) selected from a medium-sized community in the Midwest, whose responses may not reflect the experiences of African-American men in other regions of the United States; lack of self-reported or clinical data on PSA testing. Potential recall bias from study participants	Investigation of the determinants of African-American men’s health-seeking behaviour, in particular on the influence of masculine beliefs
Hill et al. 2013 [48]	USA	To understand the lived experiences and attitudes of African American men and to investigate the social and cultural behaviours, and barriers that prevented them from having prostate cancer screening	Case study using semi-structured interviews	14 AA men aged 45–64 years without a prior diagnosis of prostate cancer	Prostate cancer screening associated with homophobia which is culturally stigmatised; Perceptions of shame associated with the sexual implications of a prostate cancer diagnosis (reduced sexual performance)	Masculinity concerns; treatment side effects; sexuality concerns	Self; structural	Study findings limited to men in a small city in the United States	Need for further research to examine the effect of physicians’ gender, race and ethnicity that affect African American men’s participation in prostate cancer screening
Hughes et al. 2007 [41]	USA	To (1) investigate health behaviour, education and awareness in regard to prostate cancer; (2) explore factors that influence decisions to participate in screenings; (3) determine the feasibility of participation in a long-term follow-up study and (4) assess barriers and facilitators to participation in such studies	Qualitative study using focus groups	54 African American men aged 55–79 years who had been diagnosed with prostate cancer within their past 10 years and their female spouses (*n* = 37) aged 48–77 years	Perceptions of stigma associated with socio-cultural beliefs of imminent death following a cancer diagnosis leading to reluctance to seek help for symptoms or discuss the prostate cancer topic within social circle	Cancer belief; decision-making for diagnosis; social interactions	Structural	Potential for men and spouses to hold back sharing personal experiences in a focus group setting	Unclear
Imm et al. 2017 [49]	USA	To explore the AA prostate cancer survivorship experience and the potential unique factors contributing to the quality-of-life outcomes among AA survivors	Qualitative study using focus group	12 African American men aged 49–79 years who were treated with radical prostatectomy for prostate cancer	Feelings of: marital insecurities, compromised masculinity and fear of being “laughed at” for sexual impotence following prostate cancer treatment; leading to avoidance of social interactions on the prostate cancer subject	Spousal communication; social interactions; masculinity concerns; treatment side effect	Self; public	Results cannot be generalised to the African American community at large due to the small sample size. As with many focus groups, another limitation is the openness and willingness of participants to talk about personal experiences	Need for further research to better understand masculine norms in the African American community and their influence on support-seeking behaviours, potentially uncovering ways to reduce the social stigma associated with prostate cancer. There is need for an in-depth understanding of the adoptive patterns of African American men regarding the types of social support in order to inform intervention design for implementation
Kaninjing et al. 2018 [68]	Cameroon	To (a) explore cultural norms and beliefs that contribute to a likelihood of late-stage diagnosis of prostate cancer among men in Bamenda; (b) identify factors that influence the decision to abstain or screen for prostate cancer among men in Bamenda and (c) ascertain how men in Bamenda decide between TM and conventional medicine for prostate cancer diagnosis and treatment	Qualitative study using focus group	25 men without a prior diagnosis of prostate cancer, with average age of 59.2 years mostly married (80%) and Christians (92%)	Perceptions of prostate cancer as a taboo subject which is stigmatised and should not be discussed publicly; emanating from the cultural association of cancer with death; use of catheter for post-treatment incontinence making men to be uncomfortable to attend social events for fear of urine leakage and associated “scent”	Social interactions; masculinity concerns, treatment side effects; cancer belief	Self; structural	Development of the focus group questions was guided by existing theoretical framework that was not developed specifically for the study population and may have resulted in loss of information relevant to this population	Need for further research to fully understand and address the multifactorial issues involved in prostate cancer diagnosis, treatment and survivorship in order to eliminate the stigma and fear surrounding the disease
Kim et al. 2023 [67]	South Africa	To examine the perceptions and experiences of Black African prostate cancer patients receiving treatment at a major tertiary hospital in Johannesburg, South Africa	Qualitative Research using semi-structured interviews	28 Black South African prostate cancer patients with average age 67.6 years, mostly married (71%), mostly completed secondary school education (86%)	Feelings of obligation to avoid disclosing prostate cancer diagnosis to others to avoid being stigmatised or judged negatively by others as having lived a “reckless” life	Social interactions; illness disclosure	Public	Retrospective interviews may be subject to memory and emotional bias. Difficulty in understanding the interview questions may have limited the responses of some of the more elderly participants	Need for further studies to examine the barriers and conditions faced by men in wider healthcare settings across South Africa
King-Okoye et al. 2019 [65]	Trinidad and Tobago	To understand (i) “What are TT men’s pre-diagnosis experiences of prostate cancer?” (ii) “What are men’s beliefs and meanings about prostate cancer?” (iii) “What beliefs and interpretations guided men’s help-seeking for prostate symptoms?”	Grounded theory using face-to-face semi-structured interviews	Men diagnosed with prostate cancer, aged between 42 and 90 years	Feeling of shame due to post-treatment erectile dysfunction leading to secrecy in spousal communication and help-seeking delays; perceptions of DRE as a homosexual behaviour which is culturally stigmatised, leading to reluctance to undergo the procedure	Spousal communication; sexuality concerns; screening avoidance	Self; structural	Men experiences of pre-diagnosis were mostly represented from public health systems	Need for further research to explore the views of men attending private healthcare in order to provide a broader perspective of men’s experiences from both the public and private health systems
Malika et al. 2020 [52]	USA	To understand the knowledge, perceptions and attitudes associated with prostate cancer and how these might influence behavioural tendencies relating to preventive screening and treatment for prostate cancer	Grounded theory using focus group	33 African immigrants (18 men without a prior diagnosis of prostate cancer and 15 women) mostly (82.6%), 36 years and older, married (78.3%), and all had a college education or more	Perceptions of stigma associated with erectile dysfunction as cultural connotation with diminished masculinity leading to avoidance of social interactions of prostate cancer	Social interactions; Masculinity concerns	Structural	Potential for men and women to hold back sharing personal experiences in a focus group setting	Not stated
Malika et al. 2022 [54]	USA	To understand and compare knowledge levels and family history of the three main Black subgroups (African Americans, Caribbean immigrants, and African immigrants) in the USA and to assess the influence of knowledge on past screening behaviour and intentionality for screening in the future for prostate cancer	Concurrent mixed-methods design involving focus groups and interviews, surveys	Qualitative arm: 40 men and 21 women mostly 40 years and older (76.4%) and married (80.6%): 14 AA men and 7AA female partners, 14 Caribbean men and 9 Caribbean female partners, 13 African men and 5 African female partners Quantitative arm: 335 Black men with or without a prostate cancer diagnosis including 150 AA 134 Caribbean and 51 African immigrants; mostly married, middle class	Perceptions of stigma around diminished masculinity if men admit their health concerns within the African culture—leading to cultural avoidance of social discussions on prostate cancer illness	Social interactions; socio-cultural masculine expectations	Structural	Self-reporting of measures may have been affected by recall bias. Cross-sectional quantitative measures did not allow for causal inferences to be made. Smaller sample of African immigrant due to a shorter data collection period in comparison to the other subgroups	Further research is needed to explore the intentionality for prostate cancer screening via PSA vs DRE
Nanton and Dale 2011 [56]	UK	To identify whether and in what way ethnicity played a distinctive role in determining this experience	Qualitative study using interviews	16 Caribbean men with a median age of 72.5 years and median time since prostate cancer diagnosis of 2 years	Feelings of shame and embarrassment asking for practical support to deal with post-treatment incontinence	Masculinity concerns; treatment side effects	Self	A homogenous sample of Jamaican men due to the snowball sampling strategy adopted	Need for larger studies involving men from a more diverse range of cultural, geographical and social backgrounds
Ocho et al. 2013 [63]	Trinidad and Tobago	To explore men’s views of prostrate screening in order to identify potential implications for policy and practice to improve screening uptake	Qualitative study using focus groups (*n* = 14)	75 men aged 19–60 years during the period August 2011 to January 2012	Feelings of shame, embarrassment and discrimination associated with DRE which is a perceived as homosexual behaviour and culturally stigmatised; leading to delays in help seeking and avoidance of the subject in social interactions	Social interactions; sexuality concerns;	Self, Structural	Most participants were from public sector workplaces, with a few from private sector, businessmen or higher paid professionals whose experiences and perspectives may or may not have been different to other men as a result of their socioeconomic status	Need for further studies to compare similarities or differences in perspectives related to socioeconomic status
Ottley et al. 2019 [51]	USA	To further understand the decision-making process among Black men and the barriers they face when making treatment decisions about prostate cancer	Qualitative using Ethnography	10 Black men born in the US, living with prostate cancer at least three years, aged 50 to 78 years, with at least a high school diploma, mostly married and Christians	Feelings of humiliation from treatment side effects leading to reticence in discussing concerns with doctors	Masculinity concerns; treatment side effects	Self	Socioeconomic status of the participants not representative of Black men in the US; potential researcher bias due to role as advanced clinician, and health sciences researcher and racial assumptions	Need for further studies to examine whether Black men’s cultural views on masculinity, contribute to their prostate cancer health inequities, as well as studies which explore the decision-making processes of Black veterans diagnosed with prostate cancer in light of wider social determinants of health
Rivas et al. 2016 [57]	UK	To summarise black and minority ethnic (BME) patients’ and partners experiences of prostate cancer by examining the findings of existing qualitative studies	Systematic review	Thirteen studies of men from US and UK BME men diagnosed with prostate cancer—full demographics not provided	Perceptions of stigma associated with sexual dysfunction leading to silence around prostate cancer discussions for fear of social rejection	Social interactions; masculinity concerns; post-treatment side effects	Public	Varied settings and aims of the included may have biased the findings reported and led to the risk of stereotyping	Need for more studies on diverse ethnic groups to confirm and build on review findings and inform the design of further interventions
Seymour-Smith et al. 2016 [4]	UK	To explore men’s knowledge and understanding about PC, the barriers to screening, and their experience and treatment of PC where relevant	Qualitative study using a discursive approach with semi-structured interviews	20 African-Caribbean men (10 with prostate cancer and 10 without cancer aged 30–79 years)	Perceptions of DRE as a violation of intimate body parts which is associated with homosexual behaviour and viewed as a taboo within the African-Caribbean cultural setting	Masculinity concerns; sexuality concerns	Self; structural	An opportunistic sample of Jamaican men whose views may not be representative of all African-Caribbean men	Further research and health promotion on prostate cancer and African-Caribbean men should focus on designing information at an even younger age group in order to dispel any cultural barriers
Taljaard et al. 2020 [66]	South Africa	To explore the perceptions of black men diagnosed with prostate cancer in the public healthcare sector regarding their information needs	An exploratory-descriptive qualitative methodology using semi-structured interviews	9 Black men aged 46–76 years with locally and advanced prostate cancer	Feelings of personal embarrassment for being diagnosed with cancer which is socially stigmatised as “infectious”—perceived as “cancerous”, leading to reluctance to talk about prostate cancer publicly	Social interactions	Self; structural; Public	Interviews conducted in one setting and single point in time and patient’s information needs may change along the different phases of the cancer care continuum	Future studies to explore the perspectives of healthcare providers regarding information-giving
Vapiwala et al. 2021 [53]	USA	To better characterise stigma, beliefs and perceptions pertaining to prostate cancer among Black and Latino men and women residing in an urban community, thereby identifying potentially modifiable barriers to care	Qualitative study using focus group	34 participants: 19 Hispanics/Latinos and 15 Blacks, with equal numbers of men without a prior prostate cancer diagnosis and women (*n* = 17). Median age was 57 years (range: 18 to 85 years)	Perceptions of cultural stigmatisation of DRE and erectile dysfunction associated with prostate cancer	Masculinity concerns; Post-treatment side-effects	Structural	A small sample size from a single primary source does not represent wider Black population. Group discussion may have biased participants’ responses	There is need for further exploration of the role of gender, sexuality and religion, as well as the impact of attitudes towards providers and the medical profession on knowledge regarding stigmatised cancers such as prostate cancer
Wagland et al. 2020 [61]	UK	To explore adjustment strategies adopted by Black African (BA) and Black Caribbean (BC) men in the UK as a response to the impact of prostate cancer diagnosis and treatment effects	Qualitative arm of a larger mixed methods study using cross-sectional semi-structured interviews	14 Black African and Black Caribbean men aged between 18–42 months post-diagnosis, aged 55–85 years (median age—66)	Fear of social stigma associated with lessened masculinity following post-treatment sexual dysfunction, hindered illness disclosure to others and led to avoidance of social interactions on the prostate cancer topic	Masculinity concerns; social interactions; post-treatment side effects	Self; public	Lower response rates among BA and BC Men than the wider sample, both for the main study and subsequent Invitations to be Interviewed. The small number of participants did not allow comparison of coping strategies adopted by BA and BC men, or between men born and raised in the UK and those not	Need for further research to determine how best to convey awareness raising messages to black men
Williams et al. 2017 [50]	USA	To explore African-American prostate cancer survivors’ experiences with physical activity prescription from their physicians	Qualitative study using focus groups	12 African-American men aged 49–79 years who had completed radical prostatectomy from 7–31 months previously	Feelings of embarrassment from post-treatment urine incontinence perceived leading to avoidance to engage in post-treatment physical activity	Masculinity concerns; post-treatment side effects	Self	A small sample whose experiences may not reflect the experiences of all African-American prostate cancer survivors;	Future studies to investigate priorities and motivations to support the design of physician-led interventions to support physical activity following radical prostatectomy
Winterich et al. 2009 [44]	USA	To examine how men experience two common screenings, digital rectal exams (DREs) and colonoscopies using masculinity and health theory	Qualitative study using in-depth interviews	64 African American and White men aged 40–64 without a cancer diagnosis	Perception of digital rectal examination as a gay sexual behaviour which is culturally stigmatised within their African American community	Sexuality concerns	Structural	Perceptions of the study sample of heterosexual men from North Carolina may not reflect the concerns of gay men or men from other geographical locations regarding digital rectal examination	Larger studies are needed to determine if perceptions of homosexuality significantly affect screening rates; as well as the influence of doctor’s gender on DRE uptake
Wiseman et al. 2016 [64]	Trinidad	To determine the factors that act as barriers and facilitators to screening practice among Trinidadian men	Phenomenology using semi-structured interviews	Five men aged 40 years without a prostate cancer diagnosis	Perception of “digital rectal examination as embarrassing and emasculating”, if performed by a male doctor due to taboos associated with homosexuality. Feelings of fear and shame and social stigma related to loss of sexual function -	Masculinity concerns; sexuality concerns; post-treatment side effects	Self; structural	Only five participants recruited via healthcare professionals, church and local community may have biased the sample towards those with religious views	Need for a larger study to gain a wider understanding of Trinidadian men’s views on screening for prostate cancer
Wray et al. 2009 [45]	USA	To understand obstacles to and opportunities for improving prostate cancer communication to and within African American communities	Community-based participatory needs assessment using key informant interviews and focus groups	79 respondents (19 in key informant interviews; 32 in focus group discussants; 28 for process evaluation) -. For the focus group, 28 healthy AA men and 4 AA prostate cancer survivors; aged 40–80 years	Perceptions that prostate cancer screening via digital rectal examination carry a sense of stigma among African American men leading to screening aversion and a reluctance to talk about prostate cancer	Social interactions; screening procedure	Structural	Limitation in the evaluation of the limited number of outcome measures developed due to a lack of comparison group	Need for further research to enhance understanding on how to systematically support and promote survivors’ experience in an educational setting, for maximum impact, at reasonable cost

Data charting was done using a tested MS-Excel by two reviewers (OB with SG or BUK or OT or VN) and any arising conflicts were resolved by discussion. In line with the Arksey and O’Malley [27] framework used to guide the review, we did not perform a quality assessment of the included studies as the aim was primarily to summarise published work on stigma related to CaP in Black men and identify evidence gaps to inform future research, policy and practice. This helped to ensure an inclusive approach to addressing the review aims rather than excluding studies based on quality [34]. General characteristics of each study were extracted including: authors/year of publication, country, study aims, study design, sample, perceptions/experiences of stigma in relation to prostate cancer, the context of stigma, dimensions of stigma, study limitations and directions for future research.

#### Synthesis of Data

**Fig. 2:**
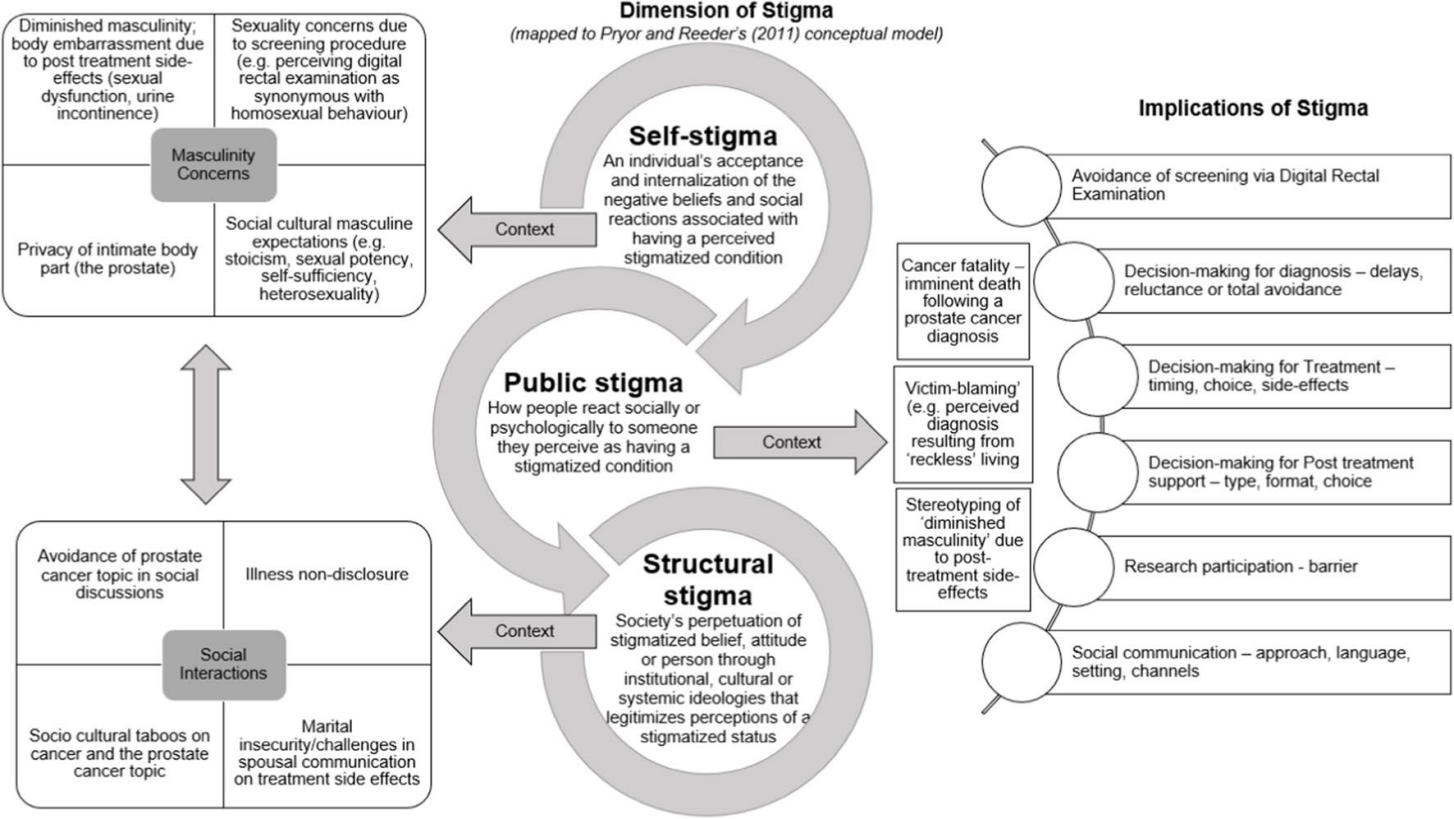
Mapping of review findings—context, dimension and implications of sigma

Data was synthesised using a descriptive qualitative content analysis [35] which involved three stages: preparation, organising and reporting [36]. In the preparation stage, a deductive approach was considered appropriate to enable data mapping to an existing theoretical framework on stigma by Pryor and Reeder [25]. In the organising phase, each included study was iteratively read to enable an in-depth understanding of where and how the data addressed the review questions. Relevant data were then extracted and mapped to the dimensions of stigma as postulated by Pryor and Reeder [25]’s conceptual model (Fig. 2) as well as the review questions to ensure clarity on the context and implications of stigma. The final stage of data reporting is discussed next in step 5. Data synthesis was done by the first reviewer (OB) and reviewed by other members of the team.

## Results

### Overview

A total of 2017 studies were retrieved from which 612 duplicates were removed. One thousand four hundred and five studies were screened for eligibility of which 1371 studies were excluded during the two-stage screening process. A final 34 eligible studies were included in the review.

### Collating, Summarising and Reporting the Evidence

Evidence was collated, summarised and reported under two broad themes to address the review questions as follows: (i) the current state of evidence on stigma related to CaP in Black men and (ii) experiences/perceptions of stigma related to CaP in Black men. The implications of review findings, recommended strategies to address CaP-related stigma within the Black community and directions for future research are enumerated later in the discussion section.

### Current state of Evidence on Stigma Related to CaP in Black Men

**Fig. 3:**
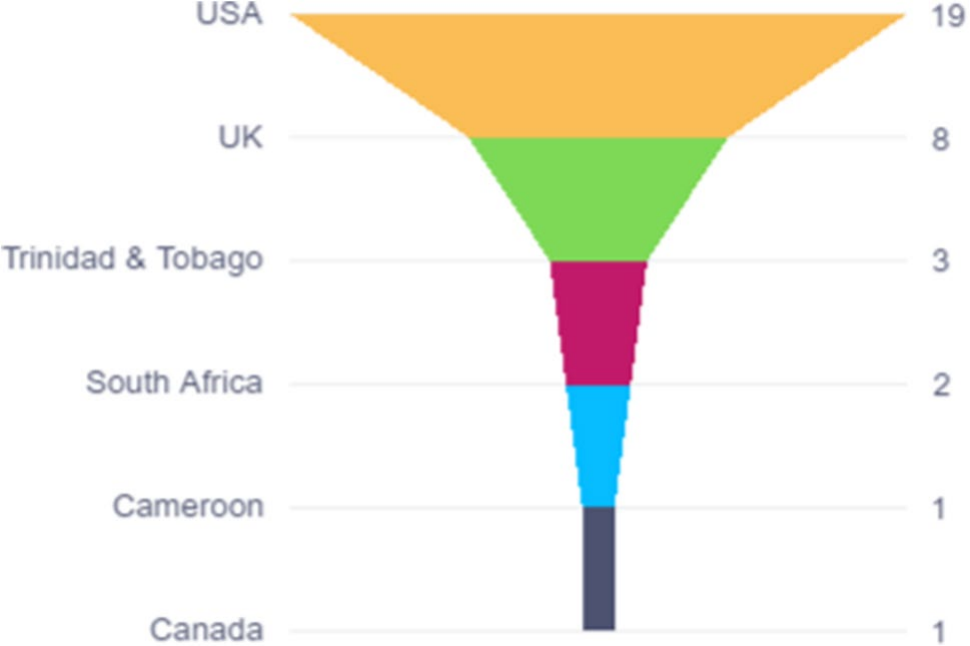
Overview of the geographical setting of included studies

Thirty-four studies conducted between 1995 and 2023 were eligible for inclusion in the review. More than half (*n* = 19) of these studies were conducted in the USA [37–55]. Eight studies were conducted in the UK [4, 56–62], three in Trinidad and Tobago [63–65], two in South Africa [66, 67], one in Cameroon [68] and one in Canada [69] (Fig. 3).

The included studies involved approximately 1867 Black men with and without a CaP diagnosis (African American, Black African, Black Caribbean, African Nova Scotian) and 145 female partners (some studies did not report sample size and ethnicity of partners) all aged 18 years and above. Detailed characteristics of the studies are presented in Table 3.

Methodologically, the included studies were of heterogeneous designs with the majority (*n* = 27) being qualitative research using focus groups (*n* = 15) [38–42, 45–47, 49, 50, 52, 53, 64, 69, 70], semi-structured interviews (*n* = 11) [4, 44, 45, 55, 56, 59, 61, 64–67], case study (*n* = 2) [48, 60] or ethnography (*n* = 1) [51]. Other study designs used were systematic review (*n* = 3) [57, 58, 62], surveys (*n* = 1) [37] and mixed methods (*n* = 2) [43, 54]. The majority of the studies involved men without a CaP diagnosis (hereunto defined as “healthy” men within the context of this review). This is reflected in the diversity of views and experiences reported across the studies. This is reported next.

### Experiences/Perceptions of Stigma Related to CaP Among Black Men

This is reported under two subthemes: lived/perceived experiences of stigma and dimensions of stigma.

#### Lived/Perceived Experiences of Stigma

Variations existed in how men described stigma as this seemed to have been shaped by their illness status (Table 3). Findings suggest that having a lived experience may have focused some men’s narratives around illness non-disclosure [37, 61–63, 68], feelings of marital insecurity [49, 65] and diminished masculinity due to post-treatment side effects such as sexual dysfunction [38, 40, 47, 48, 50, 51, 53, 55–57, 59, 61, 62, 68]. However, there were recurring themes around men’s perceptions of stigma which reverberated across the majority of the studies, regardless of illness status. Prominent among these were personal masculinity concerns and avoidance of social interactions on the CaP topic.

Masculinity concerns were mostly expressed through body embarrassment resulting from post-treatment side effects such as sexual dysfunction and urine incontinence [38, 40, 43, 47–57, 59, 61, 62, 68, 69]. There were also sexuality concerns about screening procedure via Digital Rectal Examination (DRE) which was perceived as an “assault on manhood” [Participant quote, 38], “embarrassing and emasculating” [Participant quote, [65]] and homosexual behaviour which was noted as a cultural taboo within their setting [4, 42, 44, 46, 48, 63–65]. Men in the studies further reported a perceived threat of CaP to the fulfilment of their socio-cultural masculine expectations such as stoicism, sexual potency, self-sufficiency and heterosexuality [43, 54, 55, 59]. Men expressed a need to maintain the privacy of an intimate body part (the prostate) [4, 69] as well as avoid the fear of being “laughed at” [participant quote, [49]] for sexual impotence following CaP treatment.

In many of the studies (*n* = 15), men reported stigma associated with the CaP illness itself, which is perceived as a taboo subject to be avoided in public discussions [39, 41–43, 45, 47, 52, 54, 57–59, 61, 63, 66, 68]. This was mostly attributed to perceptions of (i) imminent death following a cancer diagnosis and hospitals as “places where you go to die” [participant quote, [39]], (ii) CaP affecting men’s sexual lives [59] and a private body part “you don’t really want to reveal to people” [Participant quote, [70]], (iii) the prostate as a generational taboo which is not publicly talked about for “fear of being perceived as weak” [Participant quote, [43]], (iv) CaP viewed as an “infectious” disease in which the patient is stigmatised as “cancerous” [Participant quote, [67]], (v) avoiding being stigmatised or judged negatively by others as having lived a “reckless” life [Participant quote, [68]] and (vi) fear of social rejection [57]. In one of the studies [60], stigma was identified as a barrier to research participation as men were reluctant to invite others to participate in research on the CaP illness because they did not want to disclose their diagnosis within their wider social circle.

#### Dimensions of Stigma

Mapped to Pryor and Reeder’s [25] conceptual model of stigma, review findings showed that stigma was expressed in three out of the four dimensions identified in the model: self-stigma, public stigma and structural stigma (Fig. 2).

##### Self-stigma

Self-stigma has been described as an individual’s acceptance and internalisation of the negative beliefs and social reactions associated with having a perceived stigmatised condition [25]. In more than half of the studies (*n* = 22), participants expressed self-stigma as they seemed to accept and internalise the negative beliefs (e.g. “less of a man”) and social reactions (e.g. victim blaming) associated with being diagnosed with CaP [4, 37, 38, 40, 42, 46–51, 55, 56, 58, 59, 62–66, 68, 69]. This influenced their response to and engagement with CaP services. While men with a diagnosis of CaP in the included studies seemed to internalise personal stigma either due to the cancer diagnosis itself [66] or from post-treatment side effects [40, 49–51, 55, 56, 59, 61, 62, 65, 68]; those without a diagnosis expressed reluctance towards screening via DRE as they associated this with homosexual behaviour which was perceived as a taboo within their cultural setting [4, 38, 42, 46, 47, 58, 63, 69].

##### Public Stigma

Public stigma involves the social and psychological reactions of people to someone they perceive as having a stigmatised condition [25]. Beyond feelings of self-stigma, there were reiterations among participants across ten [38, 39, 42, 49, 57, 59, 61, 62, 66, 67] out of the 34 included studies, regarding how they perceived people within their social network (the public) generally viewed cancer and CaP in particular. Such views included perceptions of CaP as a taboo subject to be avoided in public discussions and a cancer diagnosis as imminent death [39] as well as linking CaP to men’s sexual lives which is not to be discussed in public settings [59, 61]. Men’s perceived public reactions of diminished masculinity following post-treatment sexual dysfunction further contributed to their reluctance to disclose the CaP illness diagnosis to avoid being rejected [57], labelled as “infectious” [66] or impotent [38, 62] within their social circle. This hindered some men in the included studies from accessing post-treatment psychosocial support [62]. Some men also expressed reluctance towards social interactions on prostate screening for fear of being perceived negatively [42] or “laughed at” [49] by their peers. Some men further reported feelings of marital insecurities [49] and perceptions of being judged by others as having lived a “reckless” life [67]. Whilst feelings of marital insecurity seemed to have emanated from the men themselves in one of the studies [49], there is no indication that partners stigmatise their spouse following a CaP diagnosis or that partners themselves were recipients of stigmatised attitudes from others (stigma by association).

##### Structural Stigma

Structural stigma involves the perpetuation of a stigmatised belief, attitude or person through institutional, cultural and systemic ideologies that legitimise people’s perceptions of a stigmatised status. Structural stigma was enacted in more than half of the included studies as reported by the men [*n* = 21 – 4, 37, 46, 48, 52, 54, 57, 59, 63, 65, 66, 68]. Findings from the review hinted at a systemic perception of cancer as a death sentence [39, 41, 68]; and CaP as a socio-cultural taboo which cannot be discussed freely within the Black communities [37, 42, 43, 60, 66, 68]. This in turn seemed to legitimise the reluctance of many Black men to engage in social interactions on CaP as described in many of the studies [39, 41, 42, 52, 54, 58]. There were reports in some of the studies, of cultural expectations for men to be stoic and avoid public discussions on their sexual inadequacies or concerns [43, 59]. Structural stigma was further expressed in some of the studies as systemic homophobic perceptions of DRE as homosexuality which was reported as culturally stigmatised within Black communities [4, 44–46, 48, 53, 58, 63, 65].

## Discussion

Review findings showed that CaP impacted men’s perceptions of their masculinity leading to feelings of self-stigma and public stigma. Wider research on White men [71–73] reinforces the impact of CaP on men’s perception of their masculinity post-treatment for CaP regardless of their ethnicity. Evidence highlights that masculinity concerns (as reported by men in the included studies) contribute to a higher risk of mental health issues among men with CaP compared with those in the general population [73]. For Black men, these may have been further aggravated by inherent socio-cultural hegemonic masculine expectations (such as stoicism, independence, leadership, sexual potency and virility) within their cultural settings [74–76]. Underpinned by structural stigma, Black men in the studies felt obliged to uphold cultural masculinity expectations regardless of their challenges, including a CaP diagnosis and associated sequelae.

Wider research highlights an association between stigma and reduced marital satisfaction as well as challenges in relationship communication and depression among people with a lung cancer diagnosis [77]. This resonates with evidence in the current review. However, there are indications that some Black men’s feelings of marital insecurities and communication challenges on treatment side effects may have been predicated by their masculinity concerns and self-stigma rather than public stigma [14]. Despite men’s reported feelings of marital insecurity, partners’ perspectives were not reported in any of the studies. Hence, it remains unclear the implications of stigma on partners’ lived experience of being in marital relationship with a man diagnosed with CaP within the Black cultural context. This warrants further investigation in future studies.

### Implications for Practice

Review findings showed that experiences and/or perceptions of stigma influenced how Black men responded to CaP. These included reluctance towards social discussions on the disease, presentation delays and reluctance or total avoidance of diagnostic tests especially DRE due to sexuality concerns as influenced by the perceived cultural interpretation of the procedure [39, 41, 42, 45, 46, 63, 65, 69] especially where such procedure is performed by a male doctor [64].Although the included studies did not report demographic data on men’s sexual orientation (see Table 3), it appeared they were mostly heterosexual based on their perceived cultural interpretation of DRE and feelings of embarrassment associated with the procedure. Whilst this has historically been the case (most of the studies which reported on this are older), the changing dynamics of Western society suggest a social shift in the society respecting people’s decision regarding their sexuality as a driver to advance equity, diversity and inclusion.

Arguably, inherent cultural stereotypes and beliefs may have changed over time among ethnic migrants due to acculturation to their host Western country [78]. However, Brown [79] argues that despite acculturations with those from other ethnic groups, people still substantially uphold their indigenous cultural beliefs and attitudes which may influence their social interactions and response to health messages and procedures. This has implications for structural stigma related to CaP screening via DRE and highlights the need to pay attention to how DRE is presented to Black men using avenues and languages that are culturally acceptable to them, as well as showing sensitivity to men’s sexual orientation where they are comfortable to discuss this during patient-provider consultations. As stage at diagnosis is an important predictor of survival and post-treatment quality of life [9], the importance of early presentation of CaP symptoms and diagnosis for high-risk Black men cannot be understated.

Self-stigma, public stigma and structural stigma had implications for men’s decision-making regarding treatment choice and timing [50, 51, 55, 56, 60, 62] as expressed through the psychosocial impact of treatment side effects (such as sexual dysfunction, urine incontinence, fatigue) on masculinity ideologies and functions. This accentuates the challenges of making decisions for CaP treatment among Black men seeing the complex intersection of cultural masculine expectations with a high risk of aggressive CaP, younger age at diagnosis [21, 80], treatment side effects [71], structural barriers to healthcare access [81] and patient-doctor communication issues [73].

Review findings further showed an interplay of self-stigma, public stigma and structural stigma had implications for social communications on CaP within Black communities as men expressed reluctance to either broach or engage in such discussions. Socio-cultural taboos around cancer discussions especially where intimate body parts are affected, have been reported in wider literature as a barrier to help-seeking for cancer symptoms (e.g. breast, cervical and ovarian cancers) among people across different ethnicities [82]. This reinforces the complexity of untangling the nuanced influence of cultural beliefs and gender roles and identities on the uptake and utilisation of cancer services. Members of men’s social circle attributing a CaP diagnosis to having lived a “reckless” life (public stigma) further reinforces the concept of “victim-blaming” which has been shown to have an impact on help-seeking for early diagnosis of stigmatised health conditions including lung cancer [77], HIV/AIDS [15] and Obesity [17].

### Recommended Strategies to Address Prostate Cancer-Related Stigma within Black Communities

As explained by theories of intersectionality [83, 84], tackling stigma associated with CaP can be challenging considering its intricate intersection with wider individual (e.g. gender, socio-economic status), social (e.g. culture/acculturation, masculinity), systemic (e.g. healthcare service provision) factors as well as intergenerational cultural beliefs within Black communities. This suggests that stigma cannot be tackled in isolation but there is a need to work collaboratively and in partnership with Black communities, to explore culturally appropriate approaches to negotiate self-stigma, public stigma and structural stigma related to CaP. Wider research on HIV/AIDS testing [85, 86] and COVID-19 vaccination [87] highlights the importance of partnering with local and trusted communities to co-produce effective strategies to tackle illness-related stigma and promote positive help-seeking behaviours among underserved communities.

An essential starting point to achieving this is increased public health education and awareness campaigns within Black communities, on the risk factors for CaP to debunk common misconceptions about a perceived association between lifestyle and a CaP diagnosis. Moreover, such awareness campaigns can build on the recent increase in public discussions and disclosure of the cancer illness (which used to be a taboo subject) within mainstream society as accelerated by technological advancements in cancer diagnostic and treatment procedures, the influence of social media and involvement of relatable celebrity figures to disseminate cancer messages within local communities.

Recently, innovative approaches have been introduced in some areas of the UK, for example mobile drop-in clinics for men’s health checks where urological concerns can be raised along with general health issues (The ManVan project) [88]. However, these initiatives have generally focused on encouraging early help-seeking for urological concerns (including CaP) among men within the wider population. The initiatives have not yet been evaluated to assess effectiveness in achieving early help-seeking, especially for Black men in relation to CaP. Future evaluation studies are needed in this regard, especially targeting uptake and impact on CaP outcomes in Black men. Essentially, public health messaging on CaP within Black communities should be appropriately tailored using languages, communication channels (including social media and peer champions) and timing that is best suited to men’s circumstances and where they are at in the disease pathway (e.g. pre-diagnosis, treatment stage, post-treatment). For example, to avoid notions of “victim-blaming” (public stigma) and empower men to seek timely help (including feeling confident to request a diagnostic test from their doctors), public health messaging on early diagnosis should emphasise core risk factors for CaP, including having a family history of CaP or breast cancer and being of Black African or Caribbean heritage [3]. The importance of early diagnosis and timely treatment as predictors of improved survival rates [9] should also be clearly communicated to enhance men’s understanding of the link between CaP diagnosis and clinical outcomes. This could potentially help to change their mindset towards DRE if it is clinically recommended as a diagnostic procedure [4]. Providing factual information on treatment side effects (such as sexual dysfunction) using Black peer champions that men respect and can relate with (something like a “personal buddy initiative”) can also help them to appreciate such experiences are not peculiar to them and empower them in navigating self-stigma and masculinity concerns associated with treatment side effects.

The concerns of Black men in this review, regarding the implications of DRE on their personal and social perceptions of their masculinity and sexuality cannot be ignored. This suggests the need for cultural humility in patient-provider communication on CaP diagnostic tests. There is a need for clinicians to have the necessary cultural humility (open-mindedness) to engage Black men in CaP discussions without trivialising or disrespecting their cultural values or concerns and to explain DRE as a medical procedure. The language used to describe the exam should be devoid of any sexual connotation. For example, clinicians could use the phrase “examine the prostate” not “feel the prostate” while explicitly framing the test as a medical procedure [44]. Policymakers and cancer service providers should further engage with relevant community-based organisations to explore culturally appropriate ways of addressing the concerns of Black men in relation to CaP. Embedding the three dimensions of stigma highlighted in this review within this dialogue will also help to inform the co-development and—rollout of services that meet the needs of Black men along the CaP pathway.

### Study Strengths, Limitations and Directions for Future Research

This review makes important contributions to knowledge by being the first (to the best of our knowledge) to map published literature on stigma related to CaP in Black men. The review was rigorously conducted using reproducible methods guided by validated frameworks (PRISMA guidelines and Arksey and O’Malley’s [27] framework for scoping reviews), transparent reporting and an interdisciplinary team (with subject and methodological expertise, including an information specialist). However, as early scoping searches indicated that there was very little research published before 1990 on the concepts of prostate cancer AND stigma, our search did not explicitly include outdated terms for Black people. Thus, there could be a low possibility of missing studies using outdated terms but this was minimised using supplementary searching (i.e. citation searching).

The review provides a broad overview of the experiences and/or perceptions of CaP stigma and the implications of these for policy and practice in CaP care provision and delivery for Black men. The review also identified some evidence gaps to inform future research. None of the 34 studies included in the review explicitly explored stigma as a research aim. Rather, stigma was inadvertently revealed as one of many other findings which suggests why some of the studies did not provide context as to how the stigma was expressed. There is a need for targeted research which quantitatively measures the prevalence of stigma within the context of CaP in Black men.

The majority of the studies were conducted in the US and predominantly involved men without a diagnosis of CaP. Disparities in men’s demography across diverse geographical contexts as shaped by immigration dynamics and family structures have implications for global health inequalities in CaP research, policy and practice for Black men. Hence, there is a need for more UK-based research with a focus on Black men with a diagnosis of CaP to contextually compare their experiences and/or perceptions of stigma with those not yet diagnosed with the disease.

Using longitudinal quantitative methods, future studies should investigate the influence of age on perceptions of CaP stigma and how this may vary over different generations as the influence of generational differences on perceptions of stigma remains unclear. Such longitudinal research will help to examine if there is a cultural shift in mindset across generations, what factors contribute to this and how it could potentially guide the development of future strategies to improve CaP awareness and positive action within Black communities.

The inconsistent reporting of participants’ demographics across the studies (especially on sample size and ethnicity of partners) highlights the challenges of understanding the implication of stigma on marital relationships on the partners themselves from their perspectives. In their conceptual framework, Pryor and Reeder [25] describe “stigma by association” as a dimension of stigma that involves people’s reactions to being associated with a person with a stigmatised condition and the societal reactions they receive from others because of such association. The current dearth of research on partners of Black men particularly highlights a very limited understanding of their experiences of stigma and its psychosocial implication for being in marital relationships with Black men diagnosed with CaP. With CaP now widely acknowledged as a couple’s disease [89], the unfavourable impact of men’s diagnosis on their partners’ psychosocial well-being cannot be ignored. Moreover, the critical role of partners in supporting men on their CaP journey is widely recognised [89, 90] and reinforces the need for targeted research on this population using qualitative methods to unearth their experiences/perceptions of stigma.

There is further need for intervention studies, to co-produce with Black communities, culturally appropriate resources and initiatives to demystify CaP and empower men to negotiate stigma related to the illness. Evaluation studies will further help to assess the effectiveness of such interventions in facilitating early diagnosis and improved CaP outcomes in Black men.

## Conclusion

CaP is a stigmatised disease within Black communities leading to delays in help-seeking for early diagnosis, poorer survival rates and reduced quality of life among survivors and their partners. This review aimed to map published literature on stigma related to CaP in Black men and contextually understand their experiences and/or perceptions of stigma. A complex intersection of self-stigma, public stigma and structural stigma impacted men’s perceptions of their masculinity and impacted their response to diagnostic tests (in particular DRE), treatment decision-making and social interactions on CaP. There is a need for culturally appropriate multidimensional approaches to empower Black men and their communities to negotiate stigma related to CaP. This will help to normalise social discussion CaP, encourage early help-seeking for diagnostic, treatment and post- treatment cancer services, as well as advance equity in CaP care for Black men.

## Electronic Supplementary Material

Below is the link to the electronic supplementary material.

Supplementary file1 (PDF 549 KB)

## Data Availability

The data used in this review are publicly available and included in the manuscript (see data extraction table and reference list). Full details of data availability are included in Appendix 1.

## Appendix

**Full** **Search Strategies**

**Database: Medline All from 1946**

**Platform: OVID**

**Date of last search: 3** ^**rd**^ **April 2023**

1. (black* or african* or caribbean or african-caribbean* or afro-caribbean* or african american* or (ethnic adj3 minorit*)).ti,ab,kw.
2. African Americans/ or African Continental Ancestry Group/ or Caribbean region/ or exp Blacks/
3. exp Africa/eh
4. exp Caribbean Region/eh
5. (BME or BAME).ti,ab,kw.
6. BIPOC.ti,ab,kw.
7. 1 or 2 or 3 or 4 or 5 or 6 [Black men concept]
8. exp Prostatic Neoplasms/
9. (prostat* adj2 (cancer* or neoplasm* or tumor or tumour* or malign* or carcinoma* or metasta* or oncolog*)).ti,ab,kw.
10. (Cancer Survivors/ or (cancer* adj2 survivor*).ti,ab,kw.) and prostat*.ti,ab,kw.
11. 8 or 9 or 10 [prostate cancer concept]
12. social isolation/ or ostracism/ or social marginalization/ or social stigma/
13. stereotyping/
14. social perception/ or perceived discrimination/ or social cognition/
15. Adaptation, Psychological/
16. Emotional Adjustment/
17. shame/ or embarrassment/
18. Masculinity/
19. Prejudice/
20. Social Discrimination/
21. Self Concept/
22. Bullying/
23. (stigma* or blam* or prejudice* or shame* or discrimin* or bully* or teas* or stereotyp* or misconception* or masculinity).ti,ab,kw.
24. ((public* or community or social or popular) adj perception*).ti,ab,kw,kf.
25. or/12–24 [stigma]
26. exp Erectile Dysfunction/
27. Sexual Dysfunction, Physiological/ or Sexual Dysfunctions, Psychological/
28. Ejaculation/
29. Penile Erection/
30. Sexuality/
31. sexual partners/ or spouses/
32. (sex* adj3 (dysfunction* or symptom* or health or behaviour* or behavior* or problem* or side effect* or consequence* or bother* or function* or performance*)).ti,ab,kw,kf.
33. (intimacy or impoten* or intimate relationship* or erectile dysfunction or low libido or ejaculat*).ti,ab,kw,kf.
34. or/26–33 [sexual dysfunction]

35. 7 and 11 and 25 and 34

36. 11 and 25 and 34

37. 7 and 11 and 25
38. 7 and 11 and 34

39. 35 or 36 or 37 or 38

**Database: Embase from 1974**

**Platform: OVID**

**Date of last search: 3** ^**rd**^ **April 2023**

1. (black* or african* or caribbean or african-caribbean* or afro-caribbean* or african american* or (ethnic adj3 minorit*)).ti,ab,kw.
2. black person/ or african american/ or african brazilian/ or african caribbean/ or exp "ethnic or racial aspects"/
3. (BME or BAME).ti,ab,kw.
4. BIPOC.ti,ab,kw.
5. or/1–4 [ Black men concept]
6. exp prostate cancer/
7. (prostat* adj2 (cancer* or neoplasm* or tumor or tumour* or malign* or carcinoma* or metasta* or oncolog*)).ti,ab,kw.
8. (cancer Survivor/ or (cancer* adj2 survivor*).ti,ab,kw.) and prostat*.ti,ab,kw.
9. 6 or 7 or 8 [prostate cancer concept]
10. stigma/
11. social stigma/
12. social isolation/ or social alienation/
13. ostracism/ or social exclusion/ or social rejection/
14. stereotyping/
15. masculinity/
16. shame/
17. embarrassment/
18. social discrimination/
19. bullying/
20. (stigma* or blam* or prejudice* or shame* or discrimination or bully* or teas* or stereotyp* or misconception* or masculinity).ti,ab,kw.
21. ((public* or community or social or popular) adj perception*).ti,ab,kw,kf.
22. or/10–21 [stigma]
23. impotence/ or erectile dysfunction/ or organic impotence/ or psychogenic impotence/
24. male sexual dysfunction/ or ejaculation disorder/ or potency disorder/ or premature ejaculation/
25. penis erection/
26. sexuality/
27. spouse/
28. (sex* adj3 (dysfunction* or symptom* or health or behaviour* or behavior* or problem* or side effect* or consequence* or bother* or function* or performance*)).ti,ab,kw,kf.
29. (intimacy or impoten* or intimate relationship* or erectile dysfunction or low libido or ejaculat*).ti,ab,kw,kf.
30. or/23–29 [sexual dysfunction]
31. 5 and 9 and 22 and 30
32. 5 and 9 and 30
33. 5 and 9 and 22
34. 9 and 22 and 30
35. 31 or 32 or 33 or 34

**Database: APA PsycInfo from 1967**

**Platform: OVID**

**Date of last search: 3** ^**rd**^ **April 2023**

1. blacks/ or african cultural groups/
2. (black* or african* or caribbean or african-caribbean* or afro-caribbean* or african american* or (ethnic adj3 minorit*)).ti,ab,id.
3. (BME or BAME).ti,ab,id.
4. BIPOC.ti,ab,id.
5. or/1–4 [Black men concept]
6. exp Prostate/ and exp Neoplasms/
7. (prostat* adj2 (cancer* or neoplasm* or tumor or tumour* or malign* or carcinoma* or metasta* or oncolog*)).ti,ab,id.
8. 6 or 7 [prostate cancer concept]
9. stigma/ or social perception/ or mental health stigma/ or self-stigma/ or labeling/ or prejudice/ or social acceptance/ or social discrimination/ or stereotyped attitudes/
10. shame/ or embarrassment/
11. exp Masculinity/
12. (stigma* or blam* or prejudice* or shame* or discrimin* or masculinity or bully* or teas* or stereotyp* or misconception*).ti,ab,id.
13. ((public* or community or social or popular) adj perception*).ti,ab,id.

14. or/9–13 [stigma concept]

15. sexual function disturbances/ or erectile dysfunction/ or inhibited sexual desire/ or premature ejaculation/
16. exp male orgasm/
17. libido/
18. sexual partners/
19. exp spouses/
20. (sex* adj3 (dysfunction* or symptom* or health or behaviour* or behavior* or problem* or side effect* or consequence* or bother* or function* or performance*)).ti,ab,id.
21. (intimacy or impoten* or intimate relationship* or erectile dysfunction or low libido or ejaculat*).ti,ab,id.

22. or/15–21 [sexual dysfunction concept]
23. 5 and 8 and 14 and 22
24. 5 and 8 and 22
25. 5 and 8 and 14
26. 8 and 14 and 22

27. 23 or 24 or 25 or 26

**Database: CINAHL Complete**

**Platform: EBSCOhost**

**Date of last search: 3**^**rd**^** April 2023**

**Table:**

#	Query
S1	(MH "Prostatic Neoplasms + ")
S2	TI ( (prostat* N2 (cancer* OR neoplasm* OR tumor* OR tumour* OR malign* OR carcinoma* OR metasta* OR oncolog*))) OR AB ( (prostat* N2 (cancer* OR neoplasm* OR tumor* OR tumour* OR malign* OR carcinoma* OR metasta* OR oncolog*)))
S3	TI ( ((MH “Cancer Survivors”) OR (cancer* N2 survivor*)) AND prostat*) OR AB ( ((MH “Cancer Survivors”) OR (cancer* N2 survivor*)) AND prostat*)
S4	S1 OR S2 OR S3 [prostate cancer]
S5	(MH “Black Persons + ”) OR (MH “African Americans”)
S6	TI ( (black* OR African* OR Caribbean OR African-Caribbean* OR Afro-Caribbean* OR African American* OR (ethnic N3 minorit*))) OR AB ( (black* OR African* OR Caribbean OR African-Caribbean* OR Afro-Caribbean* OR African American* OR (ethnic N3 minorit*)))
S7	TI ( BME or BAME or BIPOC) OR AB ( BME or BAME or BIPOC)
S8	S5 OR S6 OR S7 [black men]
S9	(MH “Stigma”)
S10	(MH “Ostracism”)
S11	(MH “Stereotyping”)
S12	(MH “Shame”) OR (MH “Embarrassment”)
S13	(MH “Masculinity”)
S14	(MH “Discrimination”) OR (MH “Perceived Discrimination”)
S15	(MH “Bullying”)
S16	TI ( (stigma* or blam* or prejudice* or shame* or discrimin* or bully* or teas* or stereotyp* or misconception* or masculinity)) OR AB ( (stigma* or blam* or prejudice* or shame* or discrimin* or bully* or teas* or stereotyp* or misconception* or masculinity))
S17	TI ( ((public* or community or social or popular) N1 perception*)) OR AB ( ((public* or community or social or popular) N1 perception*))
S18	(MH “Sexual Dysfunction, Male”) OR (MH “Dyspareunia”) OR (MH “Erectile Dysfunction”) OR (MH “Premature Ejaculation”) OR (MH “Sexual Desire Disorders + ”)
S19	(MH “Sexual Partners”)
S20	(MH “Spouses”)
S21	TI ( (sex* N2 (dysfunction* or symptom* or health or behaviour* or behavior* or problem* or side effect* or consequence* or bother* or function* or performance*))) OR AB ( (sex* N2 (dysfunction* or symptom* or health or behaviour* or behavior* or problem* or side effect* or consequence* or bother* or function* or performance*)))
S22	TI ( (intimacy or impoten* or intimate relationship* or erectile dysfunction or low libido or ejaculat*)) OR AB ( (intimacy or impoten* or intimate relationship* or erectile dysfunction or low libido or ejaculat*))
S23	S9 OR S10 OR S11 OR S12 OR S13 OR S14 OR S15 OR S16 OR S17 [stigma concept]
S24	S18 OR S19 OR S20 OR S21 OR S22 [sexual dysfunction]
S25	S4 AND S8 AND S23 AND S24
S26	S4 AND S8 AND S24
S27	S4 AND S8 AND S23
S28	S4 AND S23 AND S24
S29	S25 OR S26 OR S27 OR S28

**Database: Science Citation Index Expanded from 1970, Social Sciences Citation Index from 1970,**

**Platform: Web of Science Core Collection**

**Date of last search: 3** ^**rd**^ **April 2023**

**Black* or african american* or african caribbean*** (Topic) and **“prostat* cancer*”** (Topic) and **impoten* or “erectile dysfunction” or “sexual dysfunction” or “sexual side effect*”** (Topic) and **MEDLINE®** (Exclude – Database)

OR

**Black* or african american* or african caribbean*** (Topic) and **“prostat* cancer*”** (Topic) and **stigma* or shame or embarrassment or stereotype* or masculinity** (Topic)

OR

**"prostat* cancer*"** (Topic) and **stigma* or shame or embarrassment or stereotype* or masculinity** (Topic) and **impoten* or “erectile dysfunction” or “sexual dysfunction” or “sexual side effect*”** (Topic) and **MEDLINE®** (Exclude – Database)

**Database: Google Scholar**

**Date of last search: 3** ^**rd**^ **April 2023**

**Search terms**

“black men”| “african american”|African Caribbean “prostate cancer” stigma|sexual dysfunction

First 7 pages of results (sorted by relevance) cross checked against existing database results for unique studies

**Forward Citation Searches**

Studies used: 32 studies included after the initial searches were screened, 28 had PMID or DOI to be used for Citation searching in Citation Chaser on 3^rd^ April 2023.

The 28 articles were cited a total of 815 times corresponding to 650 unique article IDs. These results were downloaded into the scoping review Endnote library and duplicates were removed. After searching these remaining results for Prostate AND black men AND stigma, 58 studies remained.

**28 Articles Used for Citation Searches:**

[37–41, 43, 44, 46, 47, 49, 50, 52–54, 56–63, 65, 66, 68, 69, King-Okoye et al., 2017 (not included in the final review]

**Grey Literature**

The websites of Prostate cancer UK and Movember were searched on 3^rd^ April 2023 via Google Advanced search (limiting results to site or domain https://prostatecanceruk.org/ or https://uk.movember.com/) for stigma and black men but no additional results were identified.

## References

[1] Sung H, Ferlay J, Siegel RL, Laversanne M, Soerjomataram I, Jemal A, Bray F. Global Cancer Statistics 2020: GLOBOCAN Estimates of Incidence and Mortality Worldwide for 36 Cancers in 185 Countries. CA Cancer J Clin. 2021;2021(71):209–49. 10.3322/caac.21660 33538338

[2] Cancer Research UK. Prostate Cancer Incidence. 2022. Available at: https://www.cancerresearchuk.org/health-professional/cancer-statistics/statistics-by-cancer-type/prostate-cancer#heading-Zero. Accessed 25 Jul 2022.

[3] Prostate Cancer UK. Are you at risk? Prostate Cancer UK. 2024. Available at: Prostate Cancer Risk Factors | Prostate Cancer UK | Prostate Cancer UK. Accessed 29 May 2024.

[4] Seymour-Smith S, Brown D, Cosma G, Shopland N, Battersby S, Burton A. “Our people has got to come to terms with that”: changing perceptions of the digital rectal examination as a barrier to prostate cancer diagnosis in African-Caribbean men. Psychooncology. 2016;25(10):1183–90. 27423059 10.1002/pon.4219

[5] Mulugeta B, Williamson S, Monks R, Hack T, Beaver K. Cancer through black eyes- The views of UK based black men towards cancer: a constructivist grounded theory study. Eur J Oncol Nurs. 2017;29:8–16. 28720270 10.1016/j.ejon.2017.04.005

[6] Machirori M, Patch C, Metcalfe A. Study of the relationship between Black men, culture and prostate cancer beliefs. Cogent Med. 2018;5(1):1442636.

[7] Vapiwala N, Miller D, Laventure B, Woodhouse K, Kelly S, Avelis J, Baffic C, Goldston R, Glanz K. Stigma, beliefs and perceptions regarding prostate cancer among Black and Latino men and women. BMC Public Health. 2021;21:758. 10.1186/s12889-021-10793-x. 33879107 10.1186/s12889-021-10793-x PMC8056613

[8] Prostate Cancer UK. A Black man’s risk. London: Prostate Cancer UK. 2016. Available at: https://prostatecanceruk.org/get-involved/black-men-and-prostate-cancer/prostate-cancer-and-your-risk. Accessed 28 Jul 2022.

[9] Cancer Research UK. Survival of prostate cancer. 2022. Available at: https://www.cancerresearchuk.org/about-cancer/prostate-cancer/survival. Accessed 18 Jul 2022.

[10] van Tol-Geerdink J, Leer J, Van Oort I, Van Lin E, Weijerman P, Vergunst H, Witjes J, Stalmeier P. Quality of life after prostate cancer treatments in patients comparable at baseline. Br J Cancer. 2013;108(9):1784–9. 23612450 10.1038/bjc.2013.181 PMC3658523

[11] Bamidele O, McCaughan E. A constructivist grounded theory study on decision-making for treatment choice among Black African and Black Caribbean prostate cancer survivors. Eur J Cancer Care. 2021;2021:e13516. 10.1111/ecc.13516. 10.1111/ecc.13516 34632651

[12] Bamidele O, McGarvey H, Lagan BM, Ali N, Chinegwundoh F, McCaughan E. ‘Man in the driving seat’: a grounded theory study of the psychosocial experiences of Black African and Caribbean men treated for prostate cancer and their partners. Psychooncology. 2019. 10.1002/pon.5150. 31216078 10.1002/pon.5150

[13] Wood AW, Barden S, Terk M, Cesaretti J. The influence of stigma on the quality of life for prostate cancer survivors. J Psychosoc Oncol. 2017;35(4):451–67. 10.1080/07347332.2017.1307896. 28318410 10.1080/07347332.2017.1307896

[14] Wood A, Barden S, Terk M, Cesaretti J. Prostate cancer: the influence of stigma on quality of life and relationship satisfaction for survivors and their partners. J Psychosoc Oncol. 2019;37(3):350–66. 10.1080/07347332.2018.1489442. 30580663 10.1080/07347332.2018.1489442

[15] Turi E, Simegnew D, Fekadu G, Tolossa T, Desalegn M, Bayisa L, Mulisa D, Abajobir M. High perceived stigma among people living with HIV/AIDS in a resource limited setting in Western Ethiopia: the effect of depression and low social support. Res Palliat Care. 2021;13:389-397 389. 10.2147/HIV.S295110. 10.2147/HIV.S295110 PMC8021262 33833587

[16] Hseih E, Polo R, Qian H, Fuster-RuizdeApodaca J, M and Amo J,. Intersectionality of stigmas and health-related quality of life in people ageing with HIV in China, Europe, and Latin America. Lancet Healthy Longev. 2022;3:e206–15. 10.1016/S2666-7568(22)00003-4. 36098292 10.1016/S2666-7568(22)00003-4

[17] Fruh S, Nadglowski J, Hall H, Davis S, Crook E, Zlomke K. Obesity Stigma and Bias. Nurse Pract. 2016;12(7):425–32. 10.1016/j.nurpra.2016.05.013. 10.1016/j.nurpra.2016.05.013 PMC5386399 28408862

[18] Mazurkiewicz N, Lipowski M, Krefta J, Lipowska M. “Better if they laugh with me than at me”: the role of humor in coping with obesity-related stigma in women. Int J Environ Res Public Health. 2021;18:7974. 10.3390/ijerph18157974. 34360266 10.3390/ijerph18157974 PMC8345701

[19] Singh R, Singh B, Mahato S. Community knowledge, attitude, and perceived stigma of leprosy amongst community members living in Dhanusha and Parsa districts of Southern Central Nepal. PLoS Negl Trop Dis. 2019;13(1):e0007075. 10.1371/journal.pntd.0007075. 30633780 10.1371/journal.pntd.0007075 PMC6329495

[20] Pederson A, Earnshaw V, Clark C. Zumpf K and Burnett-Zeigler (2021) Mental health stigma among Black immigrant women in an urban setting. J Ment Health Clin Psychol. 2021;5(2):1–7. 34368814 PMC8341438

[21] Pedersen VH, Armes J, Ream E. Perceptions of prostate cancer in Black African and Black Caribbean men: a systematic review of the literature. Psychooncology. 2012;21(5):457–68. 21905156 10.1002/pon.2043

[22] Bamidele O, Alexis O, Ogunsanya M, Greenley S, Worsley A, Mitchell E. A systematic review of barriers and facilitators to access and utilisation of post treatment psychosocial support by Black men treated for prostate cancer. Support Care Cancer. 2022. 10.1007/s00520-021-06716-6. 34982226 10.1007/s00520-021-06716-6 PMC8724231

[23] Larkin D, Birtle AJ, Bradley L, Dey P, Martin CR, Pilkington M, et al. A systematic review of disease related stigmatization in patients living with prostate cancer. PLoS ONE. 2022;17(2):e0261557. 10.1371/journal.pone.0261557. 35148315 10.1371/journal.pone.0261557 PMC8836305

[24] Bos AE, Pryor JB, Reeder GD, Stutterheim SE. Stigma: advances in theory and research. Basic Appl Soc Psychol. 2013;35(1):1–9. 10.1080/01973533.2012.746147.

[25] Pryor JB, Reeder GD. HIV-related stigma. In: Hall JC, Hall BJ, Cockerell CJ, editors. HIV/AIDS in the Post-HAART Era: manifestations, treatment, and Epidemiology. Shelton, CT: PMPH-USA; 2011. p. 790–806.

[26] Doyle D, Molix L. How does stigma spoil relationships? Evidence that perceived discrimination harms romantic relationship quality through impaired self-image. J Appl Soc Psychol. 2014;44:600–10.

[27] Arksey H, O’Malley L. Scoping studies: towards a methodological framework. Int J Soc Res Methodol. 2005;8(1):19–32. 10.1080/1364557032000119616.

[28] Tricco AC, Lillie E, Zarin W, O’Brien KK, Colquhoun H, Levac D, Moher D, Peters MD, Horsley T, Weeks L, Hempel S, et al. PRISMA extension for scoping reviews (PRISMA-ScR): checklist and explanation. Ann Intern Med. 2018;169(7):467–73. 10.7326/M18-0850. 30178033 10.7326/M18-0850

[29] Peters M, Godfrey C, Khalil H, Mclnerney P, Parker D, Soares CB. Guidance for Conducting Systematic Scoping Reviews. Int J Evid Based Healthc. 2015;13:141–6. 26134548 10.1097/XEB.0000000000000050

[30] Haddaway NR, Grainger MJ, Gray CT. Citationchaser: A tool for transparent and efficient forward and backward citation chasing in systematic searching. Res Synth Methods. 2022;13(4):533–45. 10.1002/jrsm.1563. 35472127 10.1002/jrsm.1563

[31] Bramer WM, Giustini D, de Jonge GB, Holland L, Bekhuis T. De-duplication of database search results for systematic reviews in EndNote. J Med Libr Assoc. 2016;104(3):240–3. 27366130 10.3163/1536-5050.104.3.014 PMC4915647

[32] Rethlefsen ML, Kirtley S, Waffenschmidt S, et al. PRISMA-S: an extension to the PRISMA Statement for Reporting Literature Searches in Systematic Reviews. Syst Rev. 2021;10:39. 10.1186/s13643-020-01542-z. 33499930 10.1186/s13643-020-01542-z PMC7839230

[33] Covidence. World-class systematic review management. 2019. Available at: https://www.covidence.org/home. Accessed 30 Nov 2023.

[34] Tricco AC, Lillie E, Zarin W, O’Brien K, Colquhoun H, Kastner M, Levac D, Ng C, Sharpe JP, Wilson K, Kenny M, Warren R, Wilson C, Stelfox HT, Straus SE. A scoping review on the conduct and reporting of scoping reviews. BMC Med Res Methodol. 2016;9(16):15. 10.1186/s12874-016-0116-4.PMID:26857112;PMCID:PMC4746911. 10.1186/s12874-016-0116-4 PMC4746911 26857112

[35] Pollock D, Peters MDJ, Khalil H, McInerney P, Alexander L, Tricco AC, Evans C, de Moraes ÉB, Godfrey C, Pieper D, Saran A, Stern C, Munn Z. Recommendations for the extraction, analysis, and presentation of results in scoping reviews. JBI Evid Synth. 2023;21(3):520–32. 10.11124/JBIES-22-00123. 36081365 10.11124/JBIES-22-00123

[36] Elo S, Kynga H. The qualitative content analysis process. J Adv Nurs. 2008;62(1):107–15. 10.1111/j.1365-2648.2007.04569.x. 18352969 10.1111/j.1365-2648.2007.04569.x

[37] Demark-Wahnefried W, Strigo T, Catoe K, Conaway M, Brunetti M, Rimer BK, Robertson CN. Knowledge, beliefs, and prior screening behaviour among blacks and whites reporting for prostate cancer screening. Urology. 1995;46(3):346–51. 7660510 10.1016/S0090-4295(99)80218-0

[38] Blocker DE, Romocki LS, Thomas KB, Jones BL, Jackson EJ, Reid L, Campbell MK. Knowledge, beliefs and barriers associated with prostate cancer prevention and screening behaviors among African-American men. J Natl Med Assoc. 2006;98(8):1286–95. 16916126 PMC2569547

[39] Allen JD, Kennedy M, Wilson-Glover A, Gilligan TD. African-American men’s perceptions about prostate cancer: implications for designing educational interventions. Soc Sci Med. 2007;64(11):2189–200. 17399877 10.1016/j.socscimed.2007.01.007

[40] Griffith DM, Mason MA, Rodela M, Matthews DD, Tran A, Royster M, Cotton M, Eng E. A structural approach to examining prostate cancer risk for rural southern African American men. J Health Care Poor Underserved. 2007;18(4):73–101. 18065853 10.1353/hpu.2007.0121

[41] Hughes GD, Sellers DB Jr, Fraser L, Teague R, Knight BN. Prostate cancer community collaboration and partnership: education, awareness, recruitment, and outreach to southern African-American males. J Cult Divers. 2007;14(2):68–73. 19175246

[42] Fyffe D, Hudson SV, Fagan JK, Brown DR. Knowledge and barriers related to prostate and colorectal cancer prevention in underserved Black men. J Natl Med Assoc. 2008;100(10):1161–7. 18942277 10.1016/s0027-9684(15)31478-4

[43] Friedman DB, Corwin SJ, Dominick GM, Rose ID. African American men’s understanding and perceptions about prostate cancer: why multiple dimensions of health literacy are important in cancer communication. J Community Health. 2009;34(5):449–60. 19517223 10.1007/s10900-009-9167-3

[44] Winterich JA, Quandt SA, Grzywacz JG, Clark PE, Miller DP, Acuna J, Arcury TA. Masculinity and the body: how African American and White men experience cancer screening exams involving the rectum. Am J. 2009;3(4):300–9. 10.1177/1557988308321675 PMC2662681 19477742

[45] Wray RJ, McClure SM, Vijaykumar S, Smith CJ, Ivy A, Jupka K, Hess R. Changing the conversation about prostate cancer among African Americans: results of formative research. Ethn Health. 2009;14(1):27–43. 19152157 10.1080/13557850802056448

[46] Harvey IS, Alston RJ. Understanding preventive behaviors among mid-Western African-American men: a pilot qualitative study of prostate screening. J Mens Health. 2011;8(2):140–51. 21743817 10.1016/j.jomh.2011.03.005 PMC3129985

[47] Friedman DB, Thomas TL, Owens OL, Hebert JR. It takes two to talk about prostate cancer: a qualitative assessment of African American men’s and women’s cancer communication practices and recommendations. Am J. 2012;6(6):472–84. 10.1177/1557988312453478 PMC3463645 22806569

[48] Hill, Keran S. An exploratory case study of African American men on the decision making of prostate cancer screening. Diss Abstr Int: Sect B: Sci Eng. 2013;74(3-B(E)):No Pagination Specified.

[49] Imm KR, Williams F, Housten AJ, Colditz GA, Drake BF, Gilbert KL, Yang L. African American prostate cancer survivorship: exploring the role of social support in quality of life after radical prostatectomy. J Psychosoc Oncol. 2017;35(4):409–23. 28398149 10.1080/07347332.2017.1294641 PMC5683844

[50] Williams F, Imm KR, Colditz GA, Housten AJ, Yang L, Gilbert KL, Drake BF. Physician role in physical activity for African-American males undergoing radical prostatectomy for prostate cancer. Support Care Cancer. 2017;25(4):1151–8. 27999951 10.1007/s00520-016-3505-7 PMC5321695

[51] Ottley L. A Qualitative Examination of Prostate Cancer Treatment Decision-making among Black Men. The University of New Mexico ProQuest Dissertations & Theses. 2019. p. 22614931.

[52] Malika N, Ogundimu O, Roberts L, Alemi Q, Casiano C, Montgomery S. African immigrant health: prostate cancer attitudes, perceptions, and barriers. Am J Mens Health. 2020;14(4). 10.1177/1557988320945465. 10.1177/1557988320945465 PMC7444135 32815480

[53] Vapiwala N, Miller D, Laventure B, Woodhouse K, Kelly S, Avelis J, Baffic C, Goldston R, Glanz K. Stigma, beliefs and perceptions regarding prostate cancer among Black and Latino men and women. BMC Public Health. 2021;21(1):758. 33879107 10.1186/s12889-021-10793-x PMC8056613

[54] Malika N, Roberts L, Alemi Q, Casiano CA, Montgomery S. Ethnic differences among black men in prostate cancer knowledge and screening: a mixed-methods study. J Racial Ethn Health Disparities. 2022;9(3):874–85. 33783757 10.1007/s40615-021-01027-2 PMC11486286

[55] Guan A, Shim JK, Allen L, Kuo MC, Lau K, Loya Z, Brooks JD, Carroll PR, Cheng I, Chung BI, DeRouen MC, Frosch DL, Golden T, Leppert JT, Lichtensztajn DY, Lu Q, Oh DL, Sieh W, Wadhwa M, Gomez SL, Shariff-Marco S. Factors that influence treatment decisions: a qualitative study of racially and ethnically diverse patients with low- and very-low risk prostate cancer. Cancer Med. 2023;12(5):6307–17. 36404625 10.1002/cam4.5405 PMC10028041

[56] Nanton V, Dale J. ‘It don’t make sense to worry too much’: the experience of prostate cancer in African-Caribbean men in the UK. Eur J Cancer Care. 2011;20(1):62–71 (**Engl**). 10.1111/j.1365-2354.2009.01155.x 20148937

[57] Rivas C, Matheson L, Nayoan J, Glaser A, Gavin A, Wright P, Wagland R, Watson E. Ethnicity and the prostate cancer experience: a qualitative metasynthesis. Psychooncology. 2016;25(10):1147–56. 27416079 10.1002/pon.4222 PMC5096040

[58] Ahiagba P, Alexis O, Worsley A. Factors influencing black men and their partners’ knowledge of prostate cancer screening: a literature review. Br J Nurs. 2017;26(18):S14–21. 29034697 10.12968/bjon.2017.26.18.S14

[59] Bamidele O, McGarvey H, Lagan BM, Parahoo K, Chinegwundoh Mbe F, McCaughan E. “Man in the driving seat”: a grounded theory study of the psychosocial experiences of Black African and Black Caribbean men treated for prostate cancer and their partners. Psychooncology. 2019;28(8):1712–20. 31216078 10.1002/pon.5150

[60] Bamidele OO, McGarvey HE, Lagan BM, Chinegwundoh F, Ali N, McCaughan E. “Hard to reach, but not out of reach”: barriers and facilitators to recruiting Black African and Black Caribbean men with prostate cancer and their partners into qualitative research. Eur J Cancer Care. 2019;28(2):e12977 (**Engl**). 10.1111/ecc.12977 30548713

[61] Wagland R, Nayoan J, Matheson L, Rivas C, Brett J, Collaco N, Alexis O, Gavin A, Glaser AW, Watson E. Adjustment strategies amongst black African and black Caribbean men following treatment for prostate cancer: findings from the Life After Prostate Cancer Diagnosis (LAPCD) study. Eur J Cancer Care. 2020;29(1):e13183 (**Engl**). 10.1111/ecc.13183 31642565

[62] Bamidele OO, Alexis O, Ogunsanya M, Greenley S, Worsley A, Mitchell ED. Barriers and facilitators to accessing and utilising post-treatment psychosocial support by Black men treated for prostate cancer-a systematic review and qualitative synthesis. Support Care Cancer. 2022;30(5):3665–90. 34982226 10.1007/s00520-021-06716-6 PMC8724231

[63] Ocho ON, Green J. Perception of prostate screening services among men in Trinidad and Tobago. Sex Res Soc Policy: J NSRC. 2013;10(3):186–92.

[64] Wiseman C. Health beliefs and prostate screening practice among Trinidadian men. Caribb J Nurs. 2016;3(1):6–23.

[65] King-Okoye M, Arber A, Faithfull S. Beliefs that contribute to delays in diagnosis of prostate cancer among Afro-Caribbean men in Trinidad and Tobago. Psychooncology. 2019;28(6):1321–7. 30953381 10.1002/pon.5085 PMC6617795

[66] Taljaard M, Lovric GT, Makenzi AM, et al. Information Needs of Black Prostate Cancer Patients Receiving Treatment Within the South African Public Healthcare System. Oncol Ther. 2020;8:285–98. 10.1007/s40487-020-00125-1. 32856279 10.1007/s40487-020-00125-1 PMC7683621

[67] Kim AW, Lambert M, Norris SA, Mendenhall E. Perceptions and experiences of prostate cancer patients in a public tertiary hospital in urban South Africa. Ethn Health. 2023:1–16. 10.1080/13557858.2023.2174253 36746674

[68] Kaninjing E, Lopez I, Nguyen J, Odedina F, Young M. Prostate cancer screening perception, beliefs, and practices among men in Bamenda, Cameroon. Am J Mens Health. 2018;12(5):1463–72. 29658388 10.1177/1557988318768596 PMC6142138

[69] Evans J, Butler L, Etowa J, Crawley I, Rayson D, Bell DG. Gendered and cultured relations: exploring African Nova Scotians’ perceptions and experiences of breast and prostate cancer. Res Theory Nurs Pract. 2005;19(3):257–73. 16144243 10.1891/rtnp.2005.19.3.257

[70] Chambers SK, Lowe A, Hyde MK, Zajdlewicz L, Gardiner RA, Sandoe D, Dunn J. Defining young in the context of prostate cancer. Am J Mens Health. 2015;9(2):103–14. 24780936 10.1177/1557988314529991 PMC4361457

[71] McCaughan E, McKenna S, McSorley O, Parahoo K. The experience and perceptions of men with prostate cancer and their partners of the CONNECT psychosocial intervention: a qualitative exploration. J Adv Nurs. 2015;71(8):1871–82. 25818026 10.1111/jan.12648

[72] Er V, Lane JA, Martin RM, Persad R, Chinegwundoh F, Njoku V, Sutton E. Barriers and facilitators to healthy lifestyle and acceptability of a dietary and physical activity intervention among African Caribbean prostate cancer survivors in the UK: a qualitative study. BMJ Open. 2017;7(10). 10.1136/bmjopen-2017-017217. 10.1136/bmjopen-2017-017217 PMC5652511 29038181

[73] Nanton V, Appleton R, Loew J, Ahmed N, Ahmedzai S, Dale J. Men don’t talk about their health, but will they CHAT? The potential of online holistic needs assessment in prostate cancer. BJU Int. 2018;121(4):494–6. 29281846 10.1111/bju.14114

[74] Owens OL, Jackson DD, Thomas TL, Friedman DB, Hebert JR. Prostate cancer knowledge and decision making among African-American men and women in the Southeastern United States. Int J Mens Health. 2015;14(1):55–70. 26190946 PMC4505933

[75] Odimegwu C, Somefun OD. Ethnicity, gender and risky sexual behaviour among Nigerian youth: an alternative explanation. Reprod Health. 2017;14(1). 10.1186/s12978-017-0284-7. 10.1186/s12978-017-0284-7 PMC5282662 28143542

[76] Owens OL, Estrada RM, Johnson K, Cogdell M, Fried DB, Gansauer L, Kim S. I’m not a chance taker: A mixed methods exploration of factors affecting prostate cancer treatment decision-making. Ethn Health. 2019;26(8):1143–62. 10.1080/13557858.2019.1606165. 30987436 10.1080/13557858.2019.1606165 PMC7184517

[77] Dirkse D, Lamont L, Li Y, Simonič A, Bebb G, Giese-Davis J. Shame, Guilt, and Communication in Lung Cancer Patients and Their Partners. Curr Oncol. 2014;21(5):718–22. 10.3747/co.21.2034. 10.3747/co.21.2034 PMC4189577 25302043

[78] Taitt HE. Prostate cancer and Afro-Caribbean men: experiences, perceptions, and beliefs. Walden University ProQuest Dissertations & Theses. 2015. p. 3684647.

[79] Brown M. African and African-Caribbean Londoners’ experiences of cancer services: a narrative approach. Doctoral thesis, University of West London. 2014.

[80] Mulugeta B. The influence of culture on the views of Black African/African-Caribbean men living in the UK towards cancer. Doctoral thesis, University of Central Lancashire; 2014.

[81] Bamidele O, McGarvey H, Lagan BM, Ali N, Chinegwundoh F, Parahoo K, McCaughan E. Life after prostate cancer: a systematic literature review and thematic synthesis of the post-treatment experiences of Black African and Black Caribbean men. Eur J Cancer Care. 2017. 10.1111/ecc.12784. 10.1111/ecc.12784 29034575

[82] Marlow LAV, McGregor LM, Nazroo JY, Wardle J. Facilitators and barriers to help-seeking for breast and cervical cancer symptoms: a qualitative study with an ethnically diverse sample in London. Psycho-Oncology. 2014;23:749–57. 10.1002/pon.3464. 24352798 10.1002/pon.3464 PMC4282580

[83] Crenshaw K. Demarginalizing the intersection of race and sex: A black feminist critique of antidiscrimination doctrine, feminist theory and antiracist politics. University of Chicago Legal Forum, 139. 1989.

[84] Nash JC. Re-thinking intersectionality. Fem Rev. 2008;89:1–15.

[85] Thapa S, Hannes K, Cargo M, et al. Stigma reduction in relation to HIV test uptake in low- and middle-income countries: a realist review. BMC Public Health. 2018;18:1277. 10.1186/s12889-018-6156-4. 30453923 10.1186/s12889-018-6156-4 PMC6245520

[86] Lippman SA, Pettifor A, Dufour MK, Kabudula CW, et al. A community mobilisation intervention to improve engagement in HIV testing, linkage to care, and retention in care in South Africa: a cluster-randomised controlled trial. Lancet HIV. 2022;9:e617–26. 36055294 10.1016/S2352-3018(22)00192-8 PMC10617423

[87] Bologna L, Stamidis KV, Paige S, Solomon R, Bisrat F, Kisanga A, Usman S, Arale A. Why communities should be the focus to reduce stigma attached to COVID-19. Am J Trop Med Hyg. 2021;104(1):39–44. 10.4269/ajtmh.20-1329. 33258438 10.4269/ajtmh.20-1329 PMC7790080

[88] RM Partners. Man Van launched to speed up cancer diagnoses and improve healthcare access. 2022. Available at: Man Van launched to speed up cancer diagnoses and improve healthcare access - RM Partners. Accessed 4 Jun 2024.

[89] Wittmann D, Carolan M, Given B, Skolarus TA, Crossley H, An L, Palapattu G, Clark P, Montie JE. What couples say about their recovery of sexual intimacy after prostatectomy: toward the development of a conceptual model of couples’ sexual recovery after surgery for prostate cancer. J Sex Med. 2015;12(2):494–504. 25358901 10.1111/jsm.12732 PMC4373522

[90] Bamidele O, Lagan BM, McGarvey H, Wittmann D, McCaughan E. “…It might not have occurred to him that this woman that is taking care of me has some emotional needs as well…”: the Unheard Voices of Wives of Black African and Black Caribbean Men with Prostate Cancer. J Support Care Cancer. 2018. 10.1007/s00520-018-4398-4. 10.1007/s00520-018-4398-4 30112723

